# Discovery of Novel 3-Cyanopyridines as Survivin Modulators and Apoptosis Inducers

**DOI:** 10.3390/molecules25214892

**Published:** 2020-10-22

**Authors:** Rehab Sabour, Marwa F. Harras, Omkulthom Mohamed Al Kamaly, Najla Altwaijry

**Affiliations:** 1 Department of Pharmaceutical Chemistry, Faculty of Pharmacy (Girls), Al-Azhar University, Cairo 11754, Egypt; rehabsabour.pharmg@azhar.edu.eg; 2 Department of Pharmaceutical Sciences, College of Pharmacy, Princess Nourah bint Abdulrahman University, Riyadh 11461, Saudi Arabia; omalkmali@pnu.edu.sa (O.M.A.K.); naaltwaijry@pnu.edu.sa (N.A.)

**Keywords:** 3-cyanopyridine, synthesis, anticancer, cell cycle arrest, apoptosis, survivin

## Abstract

The overexpression of survivin is usually accompanied by an increased resistance of cancer cells to chemotherapeutic agents in addition to cancer aggressiveness. Consequently, survivin is considered as an attractive target to develop new promising anticancer candidates. A series of novel 3-cyanopyridine derivatives was synthesized and assessed for their cytotoxic activity against three human cancer cell lines: prostate carcinoma (PC-3), breast cancer (MDA-MB-231) and hepatocellular carcinoma (HepG2). In addition, their activities were evaluated in comparison with a standard anticancer drug 5-FU. Compounds **5c** and **5e** both exhibited promising cytotoxicity against all the tested cell lines; especially, **5e** showed better cytotoxic effect than the reference drug 5-FU. In order to evaluate the safety of these compounds, they were tested on the normal cell line WI-38, revealing their toxic selectivity toward cancer cells over normal ones. Further studies were performed in order to understand their mechanism of action; we examined the ability of our promising compounds **5c** and **5e** to induce cell cycle arrest. Both resulted in a notable induction of cell cycle arrest at the G2/M phase, along with an increase in the DNA content in the pre-G1 phase, giving us an indication of the incidence of apoptosis. **5c** and **5e** were further subjected to additional study using Annexin V-FITC assay in order to evaluate their ability to induce apoptosis. The results showed a marked increase in the early and late apoptotic cells, as well as an increase in the percentage of necrosis. Furthermore, Western blotting assay was accomplished using different concentrations of **5c** and **5e**. The results revealed a striking reduction in survivin expression through proteasome-dependent survivin degradation in addition to a decrease in the expression of some other inhibitor of apoptosis proteins (IAP) family proteins: Livin, XIAP, and C-IAP1 in a concentration-dependent manner. A docking study of **5c** and **5e** compounds in the dimerization site of survivin was also performed, showing agreement with the in vitro anti-survivin activity.

## 1. Introduction

Cancer is considered a major global disease and a leading cause of death all over the world. It is estimated that more than 1.8 million new cases of cancer will be diagnosed in 2020. The great incidence of cancer around the world encouraged searching for safer and more efficient anticancer drugs via designing new candidates possessing great selectivity on the desired target [1,2]. The growth as well as conservation of many adult tissues is attained by numerous dynamically regulated processes, including cell propagation, differentiation, and ordered programmed cell death (apoptosis) [3].

Apoptosis represents the genetic program of cell death, which is a process that is essential to maintain the cell number homeostasis of tissues and organs throughout life. The deregulation of apoptosis is considered a pathogenic aspect of different diseases. Moreover, increased cell survival is one of the tumor hallmarks [4]. There are many studies that prove the great relationship between increased resistance to chemotherapy and the declined capacity to go through apoptosis [5]. To date, chemotherapy resistance is considered an obstacle in the management of cancer, where many of the primarily responsive tumors setback and develop resistance to different chemotherapeutic agents. Accordingly, the synthesis of new agents having low liability to drug resistance mechanisms is urgently needed for the management and treatment of various forms of cancer. In the last few years, there was tangible progress in the development of cytotoxic therapies used clinically based on their ability to induce cell death programs such as apoptosis [6].

Inhibitor of apoptosis proteins (IAPs) are a family of antiapoptotic proteins that have the ability to inhibit the death of cells via modulating cellular division, cell cycle progress, and controlling signal transduction pathways. Owing to the fact that they are preferentially expressed in malignant cells and are prognostically important, they are considered as attractive therapeutic goals; hence, great efforts are in progress to develop chemical IAP inhibitors that might be valuable for the treatment of different tumors [7].

Survivin, as a member of the inhibitor of apoptosis (IAP) family, plays a vital role in both the inhibition of apoptosis and regulation of the cell cycle. It is a 16.5-kDa homodimeric cytoplasmic protein, which increases in the G2/M phase of the cell cycle followed by a rapid drop in the G1 phase; therefore, it plays an important role in mitotic cell division where it shows cell cycle-dependent expression, with a distinct accumulation at mitosis. Concerning its behavior, crystallographic studies revealed that it forms homodimers across the interface of dimerization. In addition, this dimeric structure is stabilized by the formation of non-polar connections between residues, which are mostly hydrophobic. It is overexpressed in many tumor cells accompanied by cancer cell resistance to chemotherapy and radiotherapy, cancer cell metastasis, poor prognosis, and the extremely shorter survival rates of patients. Therefore, it is considered as a cancer-specific biomarker. Hence, targeting survivin embodies an attractive strategy for the development of more selective anticancer therapeutics [8,9,10,11,12,13,14,15,16].

Recently, several strategies have been established to hinder the expression of survivin, among which the use of small molecules inhibitors is included. The development of new inhibitors that interact directly with the protein is considered a great challenge, since survivin is characterized by having no catalytic activity or natural ligands, and its role is mediated via protein-protein interactions [17].

**Figure 1:**
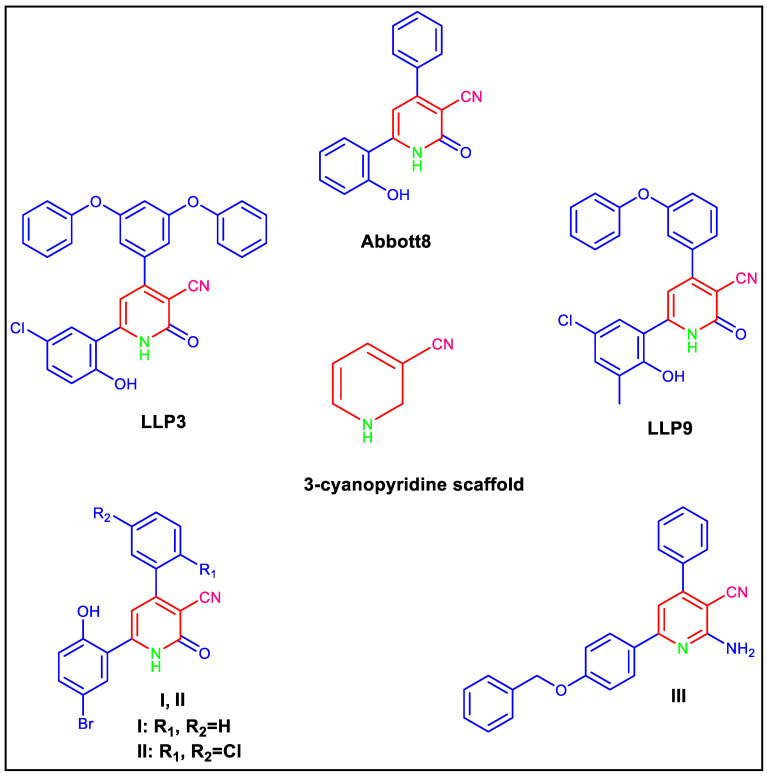
Reported 3-cyanopyridine derivatives as survivin inhibitors.

Different scaffolds have been reported that significantly suppress the expression of survivin. Interestingly, several studies in the literature have reported the effectiveness of the 3-cyanopyridine scaffold as an important survivin inhibitor. Consequently, it is considered as an encouraging template for designing a new category of chemotherapeutics [18,19]. Moreover, the dimerization interface has been reported to accommodate small molecules such as **Abbott8**. It is the prototype of a 3-cyanopyridine-based small molecule that binds at survivin′s dimerization interface [20]. In addition, structurally related analogs of **Abbott8** (**LLP3**, **LLP9**) (Figure 1) were also reported for their anti-survivin activity. The effect of **LLP3** analog on delaying mitotic progression and interference with the function of survivin in cell proliferation against two isogenic glioma cell lines was observed as it binds at the dimerization interface [21,22]. Furthermore, 3-cyano-4, 6-diaryl-2-pyridone derivatives **I** and **II** have been reported for their anti-survivin activities. It was observed that the affinity of compound **I** to survivin was enhanced via improving lipophilicity by introducing two halogen atoms to the phenyl ring at position 4 of the pyridine ring in compound **II** [23].

Encouraged by the above-mentioned findings and in continuation of our efforts [24] to discover and develop new compounds that act as effective anti-survivin chemotherapeutic agents, novel derivatives of 3-cyano pyridines were designed and synthesized to study their in vitro anti-tumor activities.

In the present study, the anti-tumor activity of the newly synthesized compounds was examined against three cell lines: prostate cancer cell line (PC-3), breast cancer cell line (MDA-MB-231), and liver cancer cell line (HepG2). Cell cycle analysis was performed along with Western blotting in order to study the mechanism of the cytotoxicity of the most active compounds.

## 2. Results and Discussion

### 2.1. Chemistry

**Scheme 1:**
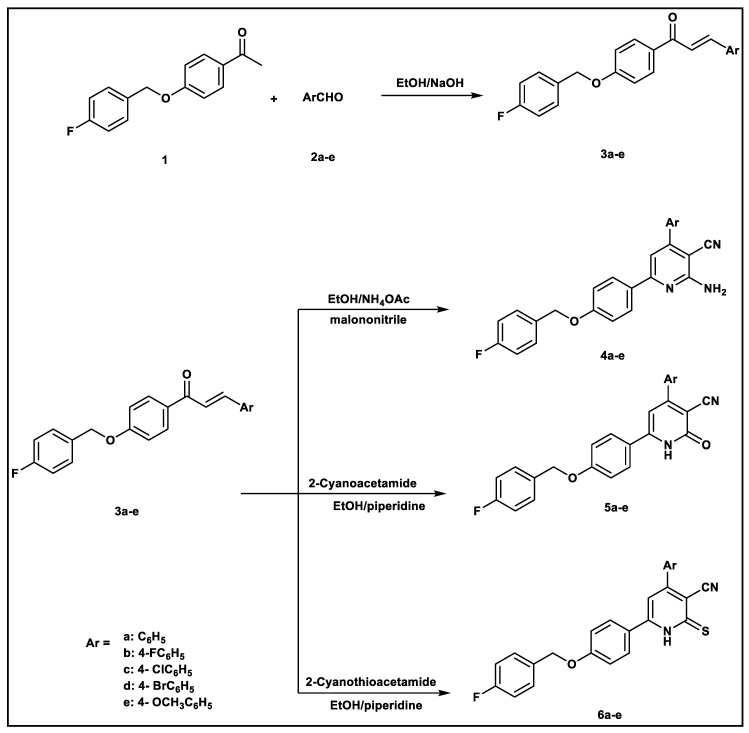
Synthetic pathway of compounds **3–6.**

The synthesis of the target 3-cyano pyridine derivatives is described in Scheme 1. The starting chalcones **3a–3e** were prepared in high yields by Claisen–Schmidt condensation [25,26] of 4-fluorobenzyloxyacetophenone with benzaldehyde/substituted benzaldehydes. The structures of these chalcones were confirmed based on their spectral and elemental analysis. The condensation reaction of chalcones **3a–3e** with malononitrile and ammonium acetate in ethanol solvent [27] yielded 2-amino cyanopyridines **4a–4e**. The IR spectrum of **4a–4e** revealed sharp bands at 2214–2219 cm^−1^ corresponding to the nitrile function and displayed bands at 3348–3206 cm^−1^ representing the amino group absorption. Additionally, as an example, the ^1^H-NMR spectrum of 4a showed singlets at 5.16 and 8.40 ppm for the O-CH_2_ and pyridine ring H-5, respectively as well as a singlet at δ 6.14 ppm exchanged with D_2_O assigned to NH_2_ protons.

On the other hand, 2-oxo-3-cyano pyridines **5a–5e** and 2-thioxo-3-cyano pyridines **6a–6e** were synthesized by the condensation of chalcones **3a–3e** with 2-cyanoacetamide and 2-cyanothioacetamide in ethanol/piperidine [28].

The proposed structures of **5a–5e** and **6a–6e** were established by microanalytical measurements. Their IR spectra presented absorption bands at 3201–3378 cm^−1^ comprising NH groups in addition to absorption bands at 2206–2222 cm^−1^ assigned to the nitrile group. Furthermore, the carbonyl function of compounds **5a–5e** appeared as strong bands in the range of 1643–1658 cm^−1^. The protons of the O-CH_2_ group of these compounds appeared as singlet signals at 5.14–5.25 ppm in their 1H-NMR spectra. Another singlet signal was seen at 6.60–6.81 ppm corresponding to H-5 of the pyridine ring for compounds **5a–5e** and at 7.47–8.03 ppm for compounds **6a–6e**. The Supplementary Material Figures S1–S36 contain the NMR charts for the synthesized compounds.

### 2.2. Biological Activity

#### 2.2.1. Cytotoxicity Evaluation

In the present work, sulforhodamine B (SRB) colorimetric assay [29,30] was done to evaluate the cytotoxic activity of the synthesized 3-cyano pyridines **4a–4e**, **5a–5e**, and **6a–6e** on three human cancer cell lines: prostate carcinoma (PC-3), breast cancer (MDA-MB-231) and hepatocellular carcinoma (HepG2) cell lines. 5-FU was used as a standard drug for comparison. The IC50 value results for the screened compounds are displayed in Table 1.

Skimming the cytotoxicity results of compounds **4a–4e** revealed poor activities with the exception of the bromo derivative **4d**, which was the only derivative that showed moderate cytotoxicity (IC_50_ = 53, 30, and 66 µM against PC-3, MDA-MB-231, and HepG2, respectively).

Concerning the 2-oxo-3-cyanopyridines **5a-5e**, significant anti-proliferative activities were observed. Compound **5e** with 4-methoxy substitution was the most active among all tested compounds. It showed twice the activity of 5-FU against the PC-3 cell line, 2.6-fold the activity against MDA-MB-231, and it was comparable to 5-FU against HepG2. In addition, the chloro derivative **5c** exhibited promising cytotoxicity that was nearly half that of 5-FU. Additionally, the rest of the 2-oxo-3-cyanopyridine derivatives revealed moderate anti-tumor activities with IC_50_ values ranging from 27.2 to 52 µM.

On the other hand, 2-thioxo-3-cyanopyridine derivatives **6a–6e** showed moderate potencies. The methoxy derivative **6e**, being the best of these compounds, displayed IC_50_ = 35.9, 23.4, and 40.3 against PC-3, MDA-MB-231, and HepG2, respectively.

Studying the SAR of our new 3-cyano pyridine derivatives revealed that in general, the 2-oxo-3-cyanopyridine scaffold is preferred for the in vitro cytotoxic activity compared to the 2-thioxo-3-cyanopyridine and 2-amino-3-cyanopyridine scaffolds. In addition, it was observed that the substituted phenyl moiety in the 4th position of the pyridine ring is more affirmative for the anti-proliferative activity than the unsubstituted phenyl group.

The cytotoxicity of the newly synthesized compounds on the normal cell line WI-38 was also evaluated in order to examine the safety of these compounds. The results revealed IC_50_ values ranging from 89.62 to 205.64 µM, revealing the safety of most compounds. Concerning the cytotoxicity of the most potent compounds **5c** and **5e**, high IC_50_ values were observed for both compounds (IC_50_ = 91.29 and 102.57 µM, respectively) and high selectivity indices (SI = 6.33, 4.56, 6.08 for **5c** and 22.99, 28.57, 17.06 for **5e**), indicating their more selective toxicity toward the cancer cells than normal cells (Table 2).

#### 2.2.2. Cell Cycle Analysis

**Figure 2:**
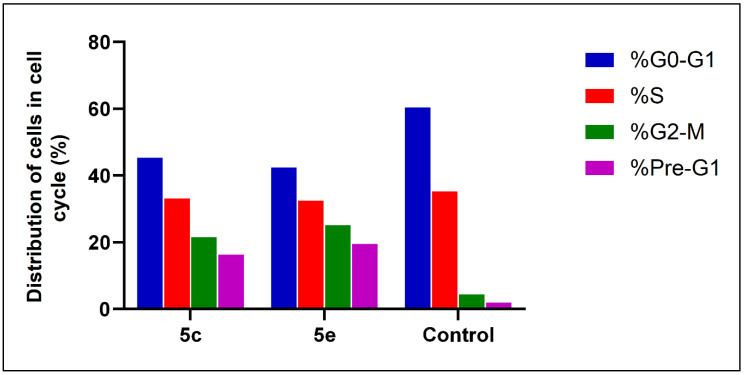
Cell cycle analysis of MDA-MB-231cells after treatment with compounds **5c** and **5e** and DMSO controls. The experiments were done in duplicates.

Survivin has been recognized to play dual roles in stimulating cell-cycle progression and in obstructing apoptosis [13]. Additionally, survivin is known to be expressed mainly in the G2/M phase of the cell cycle [8,21]. Therefore, it was significant to examine the ability of our promising compounds **5c** and **5e** to induce cell cycle arrest. Flow cytometry analysis was carried out on MDA-MB-231 cells after treatment with the IC_50_ concentrations of **5c** and **5e** for 24 h. As shown in Figure 2 and Table 3, both **5c** and **5e** caused an increase in the number of cells in the G2/M phase by 4.92 and 5.74 folds, respectively, compared to the control cells. At the same time, a decrease in the DNA content in the G0/G1 phase, and a slight decrease in the S phase cells were observed. Moreover, compounds **5c** and **5e** resulted in a remarkable increase in the pre-G1 apoptotic cells count, giving an indication of apoptosis induction. Supplementary Material Figures S37–S39 contain the cell cycle analysis for control and compounds **5c** and **5e.**

#### 2.2.3. Apoptosis Study

**Figure 3:**
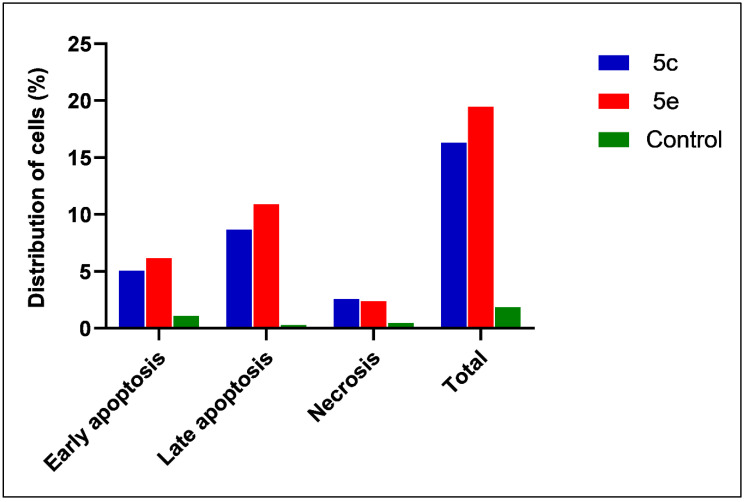
Induction of apoptosis of MDA-MB-231cells after treatment with compounds **5c** and **5e** and DMSO controls. The experiments were done in duplicates.

Survivin overexpression markedly decreased both drug-induced and spontaneous apoptosis. So, inhibiting survivin should result in increased spontaneous apoptosis in tumor cells [31]. In this study, the apoptotic potential of **5c** and **5e** was further assessed using Annexin V-FITC assay. The results are shown in Table 4 and Figure 3, displaying a significant increase in the early and late apoptotic cells, in addition to the rise in the percentage of necrosis with a total increase by 8.72 and 10.41-fold for **5c** and **5e**, respectively as compared to the control cells.

#### 2.2.4. Docking Study

Survivin is a zinc-containing protein consisting of 142 amino acids. Crystallographic studies have revealed that survivin forms homodimers across the dimerization interface that includes the amino acid residues 6–10 and 89–102. This dimeric construction is stabilized through non-polar contacts, mainly hydrophobic interactions, between residues [17,32,33]. By analysis of the dimerization interface, Leu6, Pro7, Pro8, Ala9, Trp10, Phe93, Glu94, Glu95, Leu96, Thr97, Leu98, Gly99, Phe101, and Leu102 were found to interact between the two subunits, forming a dimerization core. This core is formed essentially by the binding of Leu98 and Phe101 from one chain to the same residues of the second chain. Previous studies have indicated that this dimerization core is firmly sealed and thus is crucial for dimer stabilization. It was suggested that exposure to the hydrophobic dimeric interface can lead to conformational changes, which lead to destabilization and disruption of the dimerization core and thus may cause survivin degradation [31,34,35,36].

**Figure 4:**
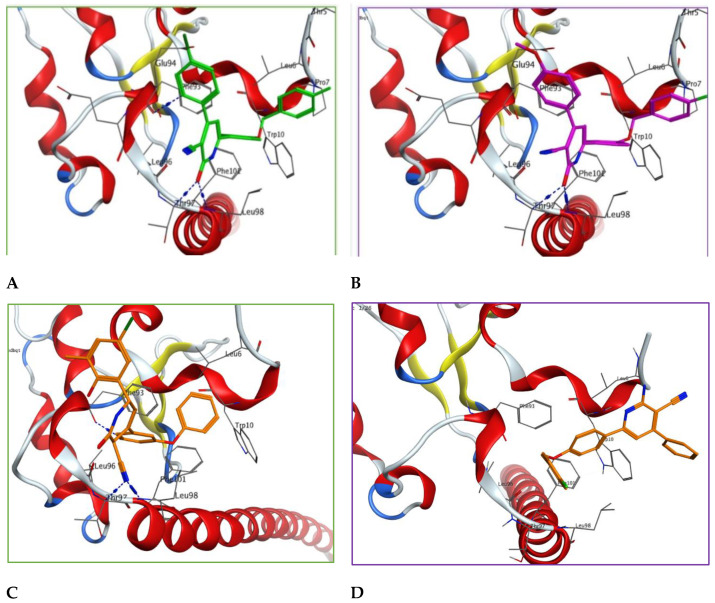
The 3D proposed binding mode of compounds **5c** (**A**), **5e** (**B**), **LLP9** (**C**), and **4a** (**D**) docked into the dimerization site of survivin.

**Figure 5:**
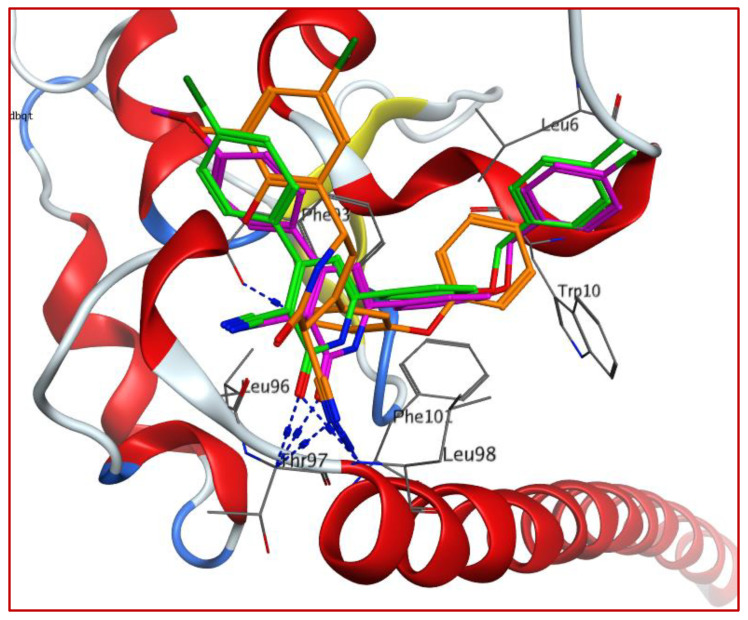
3D structure overlapping between **5c** (in green), **5e** (in purple), and **LLP9** (in orange) inside the survivin dimerization site.

Here, a molecular docking study was carried out to predict and score the poses of the protein–ligand binding using survivin monomer (PDB ID: 1E31) [32] by the MOE program [37]. The results are displayed in Table 5 and Figure 4 and Figure 5.

Results analysis revealed that the top poses of compounds **5c** and **5e** are located mainly in the dimerization core. Similar binding poses were observed for both compounds showing the following interactions in the dimerization interface: (a) hydrogen bonds between the 2-oxocyanopyridine oxygen atom and Thr97 and Leu98 residues; (b) hydrophobic interaction between the 4-aromatic moiety and Phe93 and Glu94; and (c) hydrophobic interactions between the fluorobenzyloxyphenyl moiety and the dimerization core amino acids Leu98 and Phe101 in addition to the hydrophobic residues Pro7 and Trp10.

Docking study of the ligand **LLP9** showed the same pattern as **5c** and **5e**. The 2-oxocyanopyridine cyano group formed two hydrogen bonds with Thr97 and Leu98. In addition, the phenoxy group formed hydrophobic interactions with Trp10, Leu98, and Phe101, while the phenyl moiety in the 6 position of the pyridine ring was in close contact with Phe93 and Glu94. On the other hand, the docking of compound **4a**, as an example of inactive compounds, revealed poor interaction with a lower docking score (−4.9213 kcal/mol) and a different binding mode. Although the benzyloxy phenyl group was near the Trp10 and Phe101 residues, the cyanopyridine moiety was oriented away from the dimerization site. Additionally, no hydrogen bonds were formed with the active site.

These results indicate the possibility of compounds **5c** and **5e** being oriented in the dimerization interface with the proper contacts with the essential core residues, which may be the cause of survivin inhibition.

#### 2.2.5. Western Blotting Analysis

**Figure 6:**
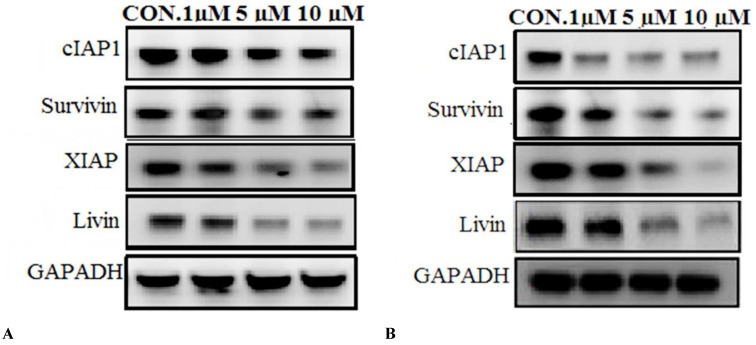
Western blot assay of MDA-MB-231 cells treated with different doses of **5c** (**A**) and **5e** (**B**) for 24 h.

The ability of the 2-oxo-3-cyano pyridines **5c** and **5e** to cause an inhibition of survivin was evaluated by the use of Western blotting analysis in MDA-MB-231cells treated with different concentrations of both compounds (1, 5, and 10 µM). As represented in Figure 4, a remarkable inhibition of survivin expression was seen in a concentration-dependent manner. Furthermore, to test the effect of these compounds on some other IAP family proteins, the Western plot of Livin, XIAP, and C-IAP1 proteins was performed (Figure 6). The results revealed that **5c** and **5e** decreased the levels of these proteins also, and that decrease is dose-dependent. The Supplementary Material Figures S40 and S41 contain western blotting analysis for **5c** and **5e**.

**Figure 7:**
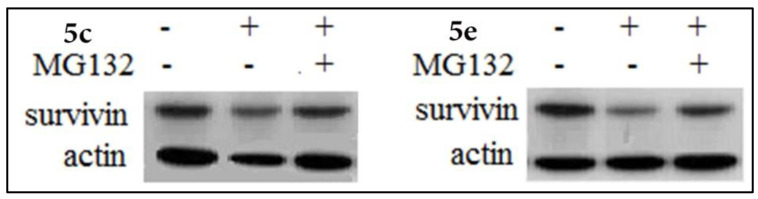
Western blot assay of MDA-MB-231 showing the effect of proteasome inhibitor MG132 on **5c** and **5e**-induced survivin degradation.

Degradation of the dimeric proteins can result from the interaction with the hydrophobic dimerization interface, which leads to conformational changes. This degradation can be mediated by the proteasome enzyme [12]. Since **5c** and **5e** were expected to inhibit survivin dimerization as shown in the docking study, we assumed that they may induce the degradation of survivin via the proteasome. Thus, a rescue experiment was performed by pre-treating MDA-MB-cells with the proteasome inhibitor MG132 prior to **5c**/**5e** treatment. As shown in Figure 7, MG132 reversed **5c**/**5e**-induced survivin loss. Thus, **5c** and **5e** likely promote survivin degradation through the proteasome.

## 3. Conclusions

A new series of 3-cyano pyridine derivatives **4a–4e**, **5a–5e**, and **6a–6e** were synthesized and their anticancer activity as survivin inhibitors against PC-3, MDA-MB-231, and HepG2 cell lines comparing to 5-FU as a reference drug was evaluated. Two compounds, **5c** and **5e**, showed promising activities against all the tested cell lines. In addition, cell cycle arrest was observed at the G2/M phase, indicating a probable induction of apoptosis. The docking study revealed that both compounds were located in the dimerization site with nearly similar binding poses. The Western blotting assay was performed in order to confirm our results, displaying a marked inhibition of different members of IAP family proteins (survivin, Livin, XIAP, and C-IAP1). Furthermore, co-treatment with the proteasome inhibitor MG132 reversed **5c/5e**-induced survivin loss.

## 4. Materials and Methods

### 4.1. Chemistry

All melting points were calculated by the Stuart SMP3 Melting Point apparatus utilizing the open capillary tube way. Elemental analysis was done at the Regional Center for Mycology and Biotechnology, Al-Azhar University. IR spectra were measured on a Shimadzu FT/IR 1650 (Perkin Elmer). ^1^H-NMR and ^13^C-NMR spectra were recorded on an Agilent Technologies 400 MHz NMR spectrophotometer using TMS as a standard, DMSO as a solvent, and D_2_O for exchangeable OH and NH protons. Mass spectra were recorded using a Shimadzu Gas Chromatograph Mass spectrometer-Qp 2010 plus. 1-(4-((4-fluorobenzyl)oxy)phenyl)ethanone (**1**) was prepared according to the reported procedure [38].

#### 4.1.1. General Procedure for the Synthesis of Compounds **3a–3e**

A mixture of compound **1** (2 mmol) and benzaldehyde/substituted benzaldehydes (2 mmol) in 40 mL of NaOH (10%) in ethanol was stirred for (6–8) h at room temperature. Then, the mixture was poured into ice-cold water that is acidified with HCl. The formed precipitate was filtered off, washed with water, and crystallized from ethanol.

**1-(4-((4-Fluorobenzyl)oxy)phenyl)-3-phenylprop-2-en-1-one (3a)**

Yield 85%; m.p. 118–120 °C; IR (KBr, cm^−1^): 3051 (CH aromatic), 2914 (CH aliphatic), 1657 (CO); ^1^H-NMR (DMSO-d6) δ (ppm): 5.22 (s, 2H, OCH_2_), 7.17 (d, 2H, aromatic H), 7.23–7.57 (m, 7H, aromatic H), 7.72 (d, 1H, olefinic H), 7.89 (d, 2H, aromatic H), 7.96 (d, 1H, olefinic H), 8.19 (d, 2H, aromatic H); ^13^C-NMR (DMSO-d6) δ (ppm): 69.30, 115.08, 115.29, 115.71, 115.92, 122.43, 129.29, 129.38, 130.60, 130.69, 131.43, 135.28, 143.70, 161.15, 162.70, 163.57, 187.83; MS, *m/z*: 331 (M^−1^, 77.42%); Anal. Calcd. For C_22_H_17_FO_2_ (332.37): C, 79.50; H, 5.16, Found: C, 79.78; H, 5.20.

**1-(4-((4-Fluorobenzyl)oxy)phenyl)-3-(4-fluorophenyl)prop-2-en-1-one (3b)**

Yield 80%; m.p. 138–140 °C; IR (KBr, cm^−1^): 3069 (CH aromatic), 2914 (CH aliphatic), 1657 (CO); ^1^H-NMR (DMSO-d6) δ (ppm): 5.23 (s, 2H, OCH_2_), 7.17 (d, 2H, aromatic H), 7.24–7.57 (m, 6H, aromatic H), 7.71 (d, 1H, olefinic H), 7.93 (d, 1H, olefinic H), 7.99 (d, 2H, aromatic H), 8.19 (d, 2H, aromatic H); ^13^C-NMR (DMSO-d6) δ (ppm): 69.30, 115.29, 115.72, 115.93, 116.28, 116.49, 122.34, 130.62, 130.71, 131.44, 131.60, 131.69, 142.47, 161.14, 162.71, 163.57, 165.03, 187.72; MS, *m/z*: 350 (M^+^, 70.95%); Anal. Calcd. For C_22_H_16_F_2_O_2_ (350.36): C, 75.42; H, 4.60, Found: C, 75.82; H, 4.81.

**3-(4-Chlorophenyl)-1-(4-((4-fluorobenzyl)oxy)phenyl)prop-2-en-1-one (3c)**

Yield 78%; m.p. 168–170 °C; IR (KBr, cm^−1^): 3067 (CH aromatic), 2914 (CH aliphatic), 1657 (CO); ^1^H-NMR (DMSO-d6) δ (ppm): 5.23 (s, 2H, OCH_2_), 7.17 (d, 2H, aromatic H), 7.23–7.55 (m, 6H, aromatic H), 7.69 (d, 1H, olefinic H), 7.94 (d, 2H, aromatic H), 7.99 (d, 1H, olefinic H), 8.19 (d, 2H, aromatic H); ^13^C-NMR (DMSO-d6) δ (ppm): 69.31, 115.32, 115.72, 115.94, 123.19, 129.41, 130.64, 130.72, 131.02, 131.49, 142.23, 161.21, 162.80, 163.62, 165.03, 187.70; MS, *m/z*: 168 [(M+2)^+^, 10.46%], 366 (M^+^, 32.10%); Anal. Calcd. For C_22_H_16_ClFO_2_ (366.81): C, 72.04; H, 4.40, Found: C, 71.85; H, 4.28.

**3-(4-Bromophenyl)-1-(4-((4-fluorobenzyl)oxy)phenyl)prop-2-en-1-one (3d)**

Yield 89%; m.p. 160–162 °C; IR (KBr, cm^−1^): 3063 (CH aromatic), 2918 (CH aliphatic), 1657 (CO); ^1^H-NMR (DMSO-d6) δ (ppm): 5.23 (s, 2H, OCH_2_), 7.17 (d, 2H, aromatic H), 7.23–7.57 (m, 6H, aromatic H), 7.69 (d, 1H, olefinic H), 7.86 (d, 2H, aromatic H), 8.00 (d, 1H, olefinic H), 8.19 (d, 2H, aromatic H); ^13^C-NMR (DMSO-d6) δ (ppm): 69.36, 115.13, 115.32, 115.72, 115.93, 123.24, 130.63, 130.71, 130.98, 131.22, 131.49, 142.23, 162.78, 163.57, 187.73; MS, *m/z*: [(M+2)^+^, 30.14%], 411 (M^+^, 35.91%); Anal. Calcd. For C_22_H_16_BrFO_2_ (411.26): C, 64.25; H, 3.92, Found: C, 64.34; H, 4.02.

**1-(4-((4-Fluorobenzyl)oxy)phenyl)-3-(4-methoxyphenyl)prop-2-en-1-one (3e)**

Yield 79%; m.p. 120–122 °C; IR (KBr, cm^−1^): 3064 (CH aromatic), 2918 (CH aliphatic), 1657 (CO); ^1^H-NMR (DMSO-d6) δ (ppm): 3.83 (s, 3H, OCH_3_), 5.23 (s, 2H, OCH_2_), 7.02 (d, 2H, aromatic H), 7.16 (d, 2H, aromatic H), 7.24–7.57 (m, 4H, aromatic H), 7.68 (d, 1H, olefinic H), 7.82 (d, 1H, olefinic H), 7.86 (d, 2H, aromatic H), 8.16 (d, 2H, aromatic H); ^13^C-NMR (DMSO-d6) δ (ppm): 55.85, 69.27, 114.86, 115.23, 115.72, 115.93, 119.90, 127.91, 130.62, 130.70, 131.19, 131.27, 131.33, 143.69, 161.14, 161.70, 162.52, 187.70; MS, *m/z*: 362 (M^+^, 24.18%); Anal. Calcd. For C_23_H_19_FO_3_ (362.39): C, 76.23; H, 5.28, Found: C, 76.14; H, 5.13.

#### 4.1.2. General Procedure for the Synthesis of Compounds **4a–4e**

A mixture of chalcones **3a–3e** (3 mmol), malononitrile (3 mmol), and ammonium acetate (9 mmol) in 20 mL of absolute ethanol was refluxed for 6 h. Then, the product was precipitated out. The reaction mixture was allowed to cool, and the formed precipitate was filtered, washed with ethanol, dried, and crystallized from ethanol.

**2-Amino-6-(4-((4-fluorobenzyl)oxy)phenyl)-4-phenylnicotinonitrile (4a)**

Yield 60%; m.p. 122–125 °C; IR (KBr, cm^−1^): 3298, 3209 (NH_2_), 3074 (CH aromatic), 2931 (CH aliphatic), 2216 (CN); ^1^H-NMR (DMSO-d6) δ (ppm): 5.16 (s, 2H, OCH_2_), 6.14 (2H, NH_2_; exchangeable with D_2_O), 7.09–8.27 (m, 13H, 13 aromatic H), 8.40 (s, 1H, CH of pyridine); ^13^C-NMR (DMSO-d6) δ (ppm): 69.08, 81.96, 114.37, 114.49, 114.57, 115.10, 115.23, 115.28, 115.42, 115.50, 115.91, 129.60, 130.61, 130.76, 131.03, 131.25, 133.60, 156.78, 160.14, 161.06, 163.47MS, *m/z*: 395 (M^+^, 9.69%); Anal. Calcd. For C_25_H_18_FN_3_O (395.43): C, 75.93; H, 4.59; N, 10.63, Found: C, 75.80; H, 4.61; N, 10.58.

**2-Amino-6-(4-((4-fluorobenzyl)oxy)phenyl)-4-(4-fluorophenyl)nicotinonitrile (4b)**

Yield 53%; m.p. 125–127 °C; IR (KBr, cm^−1^): 3325, 3217 (NH_2_), 3074 (CH aromatic), 2915 (CH aliphatic), 2217 (CN); ^1^H-NMR (DMSO-d6) δ (ppm): 5.16 (s, 2H, OCH_2_), 6.77 (2H, NH_2_; exchangeable with D_2_O), 6.94–8.30 (m, 12H, 12 aromatic H), 8.33 (s, 1H, CH of pyridine); ^13^C-NMR (DMSO-d6) δ (ppm): 69.17, 82.70, 114.96, 115.26, 115.60, 115.67, 115.80, 115.84, 117.35, 129.10, 129.17, 1130.35, 130.59, 130.62, 132.07, 132.65, 132.90, 134.17, 157.91, 159.07, 160.32, 161.15; MS, *m/z*: 413 (M^+^, 4.16%); Anal. Calcd. For C_25_H_17_F_2_N_3_O (413.42): C, 72.63; H, 4.14; N, 10.16, Found: C, 72.52; H, 4.30; N, 10.09.

**2-Amino-4-(4-chlorophenyl)-6-(4-((4-fluorobenzyl)oxy)phenyl)nicotinonitrile (4c)**

Yield 62%; m.p. 220–222 °C; IR (KBr, cm^−1^): 3348, 3206 (NH_2_), 3064 (CH aromatic), 2977 (CH aliphatic), 2219 (CN); ^1^H-NMR (DMSO-d6) δ (ppm): 5.19 (s, 2H, OCH_2_), 6.29 (2H, NH_2_; exchangeable with D_2_O), 7.09–8.15 (m, 12H, aromatic H), 8.22 (s, 1H, CH of pyridine); ^13^C-NMR (DMSO-d6) δ (ppm): 69.18, 90.94, 115.10, 115.23, 115.63, 115.68, 115.85, 115.90, 129.16, 130.53, 130.62, 130.95, 137.18, 154.27, 156.90, 160.38, 162.51; MS, *m/z*: 431 [(M+2)^+^, 3.46%], 429 (M^+^, 7.81%); Anal. Calcd. For C_25_H_17_ClFN_3_O (429.87): C, 69.85; H, 3.99; N, 9.77, Found: C, 70.00; H, 3.87; N, 9.84.

**2-Amino-4-(4-bromophenyl)-6-(4-((4-fluorobenzyl)oxy)phenyl)nicotinonitrile (4d)**

Yield 69%; m.p. 65–67 °C; IR (KBr, cm^−1^): 3338, 3212 (NH_2_), 3070 (CH aromatic), 2979 (CH aliphatic), 2219 (CN); ^1^H-NMR (DMSO-d6) δ (ppm): 5.19 (s, 2H, OCH_2_), 6.82 (2H, NH_2_; exchangeable with D_2_O), 7.12–7.56 (m, 6H, aromatic H), 7.69 (s, 1H, CH of pyridine), 7.71–8.23 (m, 6H, aromatic H); ^13^C-NMR (DMSO-d6) δ (ppm): 69.13, 91.23, 112.85, 114.78, 115.05, 115.58, 115.69, 115.90, 116.32, 128.57, 129.54, 129.88, 130.50, 130.53, 130.65, 131.02, 13.45, 156.22, 157.28, 160.79, 161.20; MS, *m/z*: 476 [(M+2)^+^, 1.76%], 474 (M^+^, 1.37%); Anal. Calcd. For C_25_H_17_BrFN_3_O (474.32): C, 63.30; H, 3.61; N, 8.86, Found: C, 63.42; H, 3.64; N, 8.93.

**2-Amino-6-(4-((4-fluorobenzyl)oxy)phenyl)-4-(4-methoxyphenyl)nicotinonitrile (4e)**

Yield 75%; m.p. 123–125 °C; IR (KBr, cm^−1^): 3302, 3240 (NH_2_), 3070 (CH aromatic), 2931 (CH aliphatic), 2214 (CN); ^1^H-NMR (DMSO-d6) δ (ppm): 3.84 (s, 3H, OCH_3_), 5.16 (s, 2H, OCH_2_), 6.81 (2H, NH_2_; exchangeable with D_2_O), 6.83–7.52 (m, 6H, aromatic H), 7.65 (s, 1H, CH of pyridine), 7.65–8.20 (m, 6H, aromatic H); ^13^C-NMR (DMSO-d6) δ (ppm): 55.83, 69.13, 91.19, 112.76, 114.10, 114.67, 115.57, 115.64, 115.85, 116.32, 128.55, 129.49, 129.89, 130.53, 130.65, 131.01, 133.42, 156.12, 157.23, 160.76, 161.17, 164.50; MS, *m/z*: 425 (M^+^, 2.92%); Anal. Calcd. For C_26_H_20_FN_3_O_2_ (425.45): C, 73.40; H, 4.74; N, 9.88, Found: C, 73.43; H, 4.60; N, 9.76.

#### 4.1.3. General Procedure for the Synthesis of Compounds **5a–5e** and **6a–6e**

A mixture of chalcones **3a–3e** (3 mmol) and cyanoacetamide/cyanothioacetamide (3 mmol) in 20 mL of absolute ethanol with few drops of piperdine was heated under reflux for 8 h, cooled, poured on ice-cold water, and acidified with HCl. Then, the precipitated solid was filtered and recrystallized from ethanol.

**6-(4-((4-Fluorobenzyl)oxy)phenyl)-2-oxo-4-phenyl-1,2-dihydropyridine-3-carbonitrile (5a)**

Yield 70%; m.p. 81–83 °C; IR (KBr, cm^−1^): 3240 (NH), 3070 (CH aromatic), 2931 (CH aliphatic), 2214 (CN), 1643 (CO); ^1^H-NMR (DMSO-d6) δ (ppm): 5.22 (s, 2H, OCH_2_), 6.76 (s, 1H, CH of pyridine), 6.84–8.18 (m, 13H, aromatic H), 9.76 (1H, NH; exchangeable with D_2_O); ^13^C-NMR (DMSO-d6) δ (ppm): 69.26, 114.05, 114.65, 114.85, 115.18, 115.23, 115.63, 115.90, 119.98, 129.48, 129.63, 130.59, 130.74, 131.16, 131.35, 143.70, 161.12, 161.69, 162.51, 162.66, 163.54; MS, *m/z*: 396 (M^+^, 31.26%); Anal. Calcd. For C_25_H_17_FN_2_O_2_ (396.41): C, 75.75; H, 4.32; N, 7.07, Found: C, 75.68; H, 4.27; N, 7.17.

**6-(4-((4-Fluorobenzyl)oxy)phenyl)-4-(4-fluorophenyl)-2-oxo-1,2-dihydropyridine-3-carbonitrile (5b)**

Yield 55%; m.p. 178–179 °C; IR (KBr, cm^−1^): 3201 (NH), 3070 (CH aromatic), 2931 (CH aliphatic), 2222 (CN), 1658 (CO); ^1^H-NMR (DMSO-d6) δ (ppm): 5.20 (s, 2H, OCH_2_), 6.81 (s, 1H, CH of pyridine), 7.01–7.92 (m, 12H, aromatic H), 9.83 (1H, NH; exchangeable with D_2_O); ^13^C-NMR (DMSO-d6) δ (ppm): 69.37, 115.10, 115.49, 115.63, 115.90, 116.15, 116.36, 127.35, 129.99, 130.32, 130.41, 130.60, 130.73, 130.94, 131.23, 131.32, 161.11, 161.18, 162.49, 162.72, 163.57; MS, *m/z*: 414 (M^+^, 19.56%); Anal. Calcd. For C_25_H_16_F_2_N_2_O_2_ (414.40): C, 72.46; H, 3.89; N, 6.76, Found: C, 72.41; H, 4.01; N, 6.84.

**4-(4-Chlorophenyl)-6-(4-((4-fluorobenzyl)oxy)phenyl)-2-oxo-1,2-dihydropyridine-3-carbonitrile (5c)**

Yield 75%; m.p. 122–2124 °C; IR (KBr, cm^−1^): 3283 (NH), 3070 (CH aromatic), 2931 (CH aliphatic), 2222 (CN), 1651 (CO); ^1^H-NMR (DMSO-d6) δ (ppm): 5.19 (s, 2H, OCH_2_), 6.80 (s, 1H, CH of pyridine), 7.01–8.20 (m, 12H, aromatic H), 9.86 (1H, NH; exchangeable with D_2_O); ^13^C-NMR (DMSO-d6) δ (ppm): 69.35, 115.16, 115.31, 115.66, 115.69, 115.90, 127.32, 128.92, 129.29, 129.39, 130.12, 130.52, 130.71, 131.02, 131.47, 135.45, 161.12, 161.25, 162.56, 162.76, 163.54; MS, *m/z*: 432 [(M+2)^+^, 30.87%], 430 (M^+^, 100.00%); Anal. Calcd. For C_25_H_16_ClFN_2_O_2_ (430.86): C, 69.69; H, 3.74; N, 6.50, Found: C, 69.86; H, 3.70; N, 6.74.

**4-(4-Bromophenyl)-6-(4-((4-fluorobenzyl)oxy)phenyl)-2-oxo-1,2-dihydropyridine-3-carbonitrile (5d)**

Yield 63%; m.p. 160–162 °C; IR (KBr, cm^−1^): 3218 (NH), 3040 (CH aromatic), 2929 (CH aliphatic), 2217 (CN), 1643 (CO); ^1^H-NMR (DMSO-d6) δ (ppm): 5.19 (s, 2H, OCH_2_), 6.60 (s, 1H, CH of pyridine), 7.14–7.90 (m, 12H, aromatic H), 9.83 (1H, NH; exchangeable with D_2_O); ^13^C-NMR (DMSO-d6) δ (ppm): 69.35, 115.42, 115.63, 115.67, 115.90, 128.97, 129.53, 129.95, 130.46, 130.54, 130.60, 130.85, 132.20, 132.31, 137.28, 151.09, 159.95, 161.17, 163.49, 164.20; MS, *m/z*: 477 [(M+2)^+^, 22.29%], 475 (M^+^, 31.82%); Anal. Calcd. For C_25_H_16_BrFN_2_O_2_ (475.31): C, 63.17; H, 3.39; N, 5.89, Found: C, 63.08; H, 3.22; N, 5.95.

**6-(4-((4-Fluorobenzyl)oxy)phenyl)-4-(4-methoxyphenyl)-2-oxo-1,2-dihydropyridine-3-carbonitrile (5e)**

Yield 77%; m.p. 90–92 °C; IR (KBr, cm^−1^): 3234 (NH), 3078 (CH aromatic), 2939 (CH aliphatic), 2214 (CN), 1651 (CO); ^1^H-NMR (DMSO-d6) δ (ppm): 3.83 (s, 3H, OCH_3_), 5.25 (s, 2H, OCH_2_), 6.83 (s, 1H, CH of pyridine), 7.01–8.15 (m, 12H, aromatic H), 9.76 (1H, NH; exchangeable with D_2_O); ^13^C-NMR (DMSO-d6) δ (ppm): 55.84, 69.27, 114.85, 115.23, 115.64, 115.70, 115.95, 119.98, 130.49, 130.60, 130.68, 131.16, 131.25, 131.39, 161.18, 161.21, 161.78, 162.49, 163.55; MS, *m/z*: 426 (M^+^, 24.92%); Anal. Calcd. For C_25_H_19_FN_2_O_3_ (426.44): C, 73.23; H, 4.49; N, 6.57, Found: C, 73.09; H, 4.35; N, 6.84.

**6-(4-((4-Fluorobenzyl)oxy)phenyl)-4-phenyl-2-thioxo-1,2-dihydropyridine-3-carbonitrile (6a)**

Yield 73%; m.p. 110–112 °C; IR (KBr, cm^−1^): 3378 (NH), 3059 (CH aromatic), 2932 (CH aliphatic), 2213 (CN); ^1^H-NMR (DMSO-d6) δ (ppm): 5.14 (s, 2H, OCH_2_), 5.85 (1H, NH; exchangeable with D_2_O), 7.06–7.58 (m, 7H, aromatic H), 7.95 (s, 1H, CH of pyridine), 8.03–8.28 (m, 6H, aromatic H); ^13^C-NMR (DMSO-d6) δ (ppm): 69.35, 115.10, 115.67, 115.82, 128.77, 128.87, 129.03, 129.16, 129.29, 129.91, 130.45, 130.53, 133.50, 152.79, 159.82, 160.71, 161.08, 163.43; MS, *m/z*: 412 (M^+^, 24.66%); Anal. Calcd. For C_25_H_17_FN_2_OS (412.48): C, 72.80; H, 4.15; N, 6.79, Found: C, 72.92; H, 4.23; N, 6.82.

**6-(4-((4-Fluorobenzyl)oxy)phenyl)-4-(4-fluorophenyl)-2-thioxo-1,2-dihydropyridine-3-carbonitrile (6b)**

Yield 53%; m.p. 114–116 °C; IR (KBr, cm^−1^): 3303 (NH), 3072 (CH aromatic), 2935 (CH aliphatic), 2213 (CN); ^1^H-NMR (DMSO-d6) δ (ppm): 5.21 (s, 2H, OCH_2_), 6.75 (1H, NH; exchangeable with D_2_O), 7.14–7.70 (m, 8H, aromatic H), 7.85 (s, 1H, CH of pyridine), 8.17–8.27 (m, 4H, aromatic H); ^13^C-NMR (DMSO-d6) δ (ppm): 69.09, 115.48, 115.63, 115.87, 115.99, 116.24, 116.45, 129.44, 129.78, 130.48, 130.54, 131.52, 131.73, 131.82, 133.46, 153.60, 161.07, 163.50, 165.28, 167.54; MS, *m/z*: 430 (M^+^, 83.68%); Anal. Calcd. For C_25_H_16_F_2_N_2_OS (430.47): C, 69.75; H, 3.75; N, 6.51, Found: C, 69.64; H, 3.51; N, 6.60.

**4-(4-Chlorophenyl)-6-(4-((4-fluorobenzyl)oxy)phenyl)-2-thioxo-1,2-dihydropyridine-3-carbonitrile (6c)**

Yield 70%; m.p. 92–93 °C; IR (KBr, cm^−1^): 3327 (NH), 3068 (CH aromatic), 2935 (CH aliphatic), 2215 (CN); ^1^H-NMR (DMSO-d6) δ (ppm): 5.09 (1H, NH; exchangeable with D_2_O), 5.19 (s, 2H, OCH_2_), 6.98–7.85 (m, 8H, aromatic H), 8.03 (s, 1H, CH of pyridine), 8.07–8.27 (m, 4H, aromatic H); ^13^C-NMR (DMSO-d6) δ (ppm): 69.13, 115.50, 115.64, 115.88, 129.18, 129.37, 129.50, 129.65, 129.76, 130.51, 130.57, 131.08, 131.22, 133.21, 133.44, 158.62, 161.08, 161.21, 161.33, 163.50; MS, *m/z*: 448 [(M+2)^+^, 4.96%], 446 (M^+^, 16.29%); Anal. Calcd. For C_25_H_16_ClFN_2_OS (446.92): C, 67.19; H, 3.61; N, 6.27, Found: C, 69.20; H, 3.55; N, 6.34.

**4-(4-Bromophenyl)-6-(4-((4-fluorobenzyl)oxy)phenyl)-2-thioxo-1,2-dihydropyridine-3-carbonitrile (6d)**

Yield 70%; m.p. 75–77 °C; IR (KBr, cm^−1^): 3305 (NH), 3062 (CH aromatic), 2939 (CH aliphatic), 2206 (CN); ^1^H-NMR (DMSO-d6) δ (ppm): 5.18 (s, 2H, OCH_2_), 7.13–7.26 (m, 4H, aromatic H), 7.47 (s, 1H, CH of pyridine), 7.52–8.19 (m, 8H, aromatic H), 8.59 (1H, NH; exchangeable with D_2_O); ^13^C-NMR (DMSO-d6) δ (ppm): 69.11, 115.13, 115.33, 115.50, 115.70, 115.88, 123.29, 129.46, 129.51, 130.50, 130.61, 131.26, 131.53, 132.09, 133.17, 155.81, 160.75, 162.52, 162.59, 162.75; MS, *m/z*: 493 [(M+2)^+^, 25.16%], 491 (M^+^, 28.39%); Anal. Calcd. For C_25_H_16_BrFN_2_OS (491.37): C, 61.11; H, 3.28; N, 5.70, Found: C, 61.22; H, 3.35; N, 5.75.

**6-(4-((4-Fluorobenzyl)oxy)phenyl)-4-(4-methoxyphenyl)-2-thioxo-1,2-dihydropyridine-3-carbonitrile (6e)**

Yield 58%; m.p. 183–185 °C; IR (KBr, cm^−1^): 3377 (NH), 3078 (CH aromatic), 2939 (CH aliphatic), 2214 (CN); ^1^H-NMR (DMSO-d6) δ (ppm): 3.85 (s, 3H, OCH_3_), 5.19 (s, 2H, OCH_2_), 5.27 (1H, NH; exchangeable with D_2_O), 7.04–7.54 (m, 8H, aromatic H), 7.70 (s, 1H, CH of pyridine), 8.02–8.21 (m, 4H, aromatic H); ^13^C-NMR (DMSO-d6) δ (ppm): 55.86, 69.10, 114.59, 114.70, 114.79, 115.38, 115.64, 115.87, 129.59, 130.47, 130.55, 130.71, 130.93, 133.60, 157.12, 160.75, 161.08, 161.21, 163.53, 163.61; MS, *m/z*: 442 (M^+^, 15.54%); Anal. Calcd. For C_26_H_19_FN_2_O_2_S (442.50): C, 70.57; H, 4.33; N, 6.33, Found: C, 70.50; H, 4.19; N, 6.22.

### 4.2. Biological Activity

#### 4.2.1. Drugs and Drug Treatments

Compounds under investigation were dissolved in DMSO to make a stock solution. Solutol^®^ HS15 is a nonionic solubilizer that consists of polyglycol mono- and di-esters of 12-hydroxystearic acid with approximately 30% of free polyethylene glycol for making the injection solutions.

#### 4.2.2. Cytotoxicity Evaluation

Human cancer cells were obtained from Nawah Scientific Inc. (Mokatam, Cairo, Egypt). Cells were maintained in DMEM media containing 100mg/mL streptomycin, 100 units/mL penicillin, and 10% heat-inactivated fetal bovine serum in humified, 5% (*v*/*v*) CO_2_ atmosphere at 37 °C.

Cell viability was estimated using the SRB assay. Aliquots of 100 µL cell suspension (5 × 10^3^ cells) in 96-well plates were incubated in complete media for 24 h. Cells were treated with a second aliquot of 100 µL media containing various concentrations of the tested compounds (0.01, 0.1, 1, 10, and 100 μM). After 72 h, cells were fixed by replacing media with 15 µL of 10% TCA and then incubated at 4 °C for 1 h. After that, TCA solution was removed, and the cells were washed 5 times with distilled water. Aliquots of 70 µL SRB solution (0.4% *w*/*v*) were added and incubated in a dark place at room temperature for 10 min. Plates were washed 3 times with 1% acetic acid and allowed to air dry overnight. Then, 150 µL of TRIS (10 mM) was added to dissolve protein-bound SRB stain. The absorbance was measured at 540 nm using BMG LABTECH^®^-FLUO star Omega microplate reader (Ortenberg, Germany).

#### 4.2.3. Cell Cycle Analysis

MDA-MB-231 cells (density = 4 × 10^6^ cells by T 75 flasks) were treated with **5c** and **5e** at their IC50 concentrations for 24 h. After that, cells were collected by trypsin and washed in saline buffered with phosphate. Then, they were fixed using ice-cold alcohol. Afterwards, cells were stained by a Cycle TESTTM PLUS DNA Reagent Kit (BD Biosciences, San Jose, CA, USA) according to the manufacturer’s directions. The distribution of the cells in the cell cycle was calculated using a FACS Calibur flow cytometer (BD Biosciences, San Jose, CA, USA).

#### 4.2.4. Apoptotic Analysis

MDA-MB-231 cells were plated in 24-well tissue culture plates. After 24 h, compounds **5c** and **5e** were added at their IC_50_ concentrations. After 48 h, cells that floated were collected from the medium. The adherent cells were separated using 0.05% trypsin. After that, a 10% FBS medium was added for inactivating trypsin. Following slow pipetting, the centrifugation of cells was done for 5 min at 1500× *g*. Then, the supernatant was discarded, and cells were then stained with double annexin V-fluorescein isothiocyanate in addition to propidium iodide (PI) staining following the manufacturer’s directions. Analysis of the cells was investigated using a FACScan flow cytometer. 

#### 4.2.5. Western Blotting Analysis

MDA-MB-231 cancer cells were incubated with 5c and 5e at different concentrations (1, 5, and 10 µM) for 48 h. Afterwards, the medium was detached, and the cells were washed using PBS (10 mmol/L, pH 7.4). Then, the cells were incubated on cold lysis buffer (50 mmol/L Tris–HCl, 150 mmol/L NaCl, 1 mmol/L EGTA, 1 mmol/L EDTA, 20 mmol/L NaF, 100 mmol/L Na3VO4, 0.5% NP40, 1% Triton X-100, 1 mmol/L PMSF (pH 7.4)) and a freshly added cocktail inhibitor (Inhibitor Cocktail Set III, Calbiochem, La Jolla, CA) above ice for 30 min. Then, MDA-MB-231 cells were collected and centrifuged at 12,000× *g* for 15 min.

XIAp, Survivin, Livin, and cIAP1anti-mouse, anti-rabbit secondary antibodies, and GAPDH were purchased from Sigma-Aldrich, whereas the gel was obtained from BioRad. Polyacrylamide gels were moved to a nitrocellulose membrane in order to block in buffer (7% nonfat dry milk/1% Tween 20; in 20 mmol/L TBS (pH 7.6)) for 1 h and incubated for 2 h at room temperature with the suitable primary antibody in blocking buffer or overnight at 4 °C; then, they were incubated with secondary antibody HRP conjugate. Finally, the blots analysis was done using the imaging system [39,40,41].

### 4.3. Molecular Docking Study

Molecular docking analysis was performed using MOE-Dock 2014.09. The chemical structures of **5c** and **5e** were sketched using Chemdraw and then imported to MOE, where they were exposed to energy minimization and conformational search. Now, their 3D conformers were docked into the survivin protein dimerization site (PDB code: 1E31). In the docking process, London dG was reserved for ranking and the GBVI/WSA dG was used for the scoring of the generated poses. Analysis and visualization of the results were performed using the “Ligand Interactions” tool to display the binding modes of these compounds in the survivin active site.

## Figures and Tables

**Table 1:** Cytotoxicity of compounds **4–6** and 5-FU evaluated in different human cancer cells. ^a^

Compound		IC_50_ (µM)	
	**PC-3**	**MDA-MB-231**	**HepG2**
**4a**	>100	>100	109 ± 0.35
**4b**	>100	>100	>100
**4c**	>100	>100	62 ± 0.61
**4d**	53 ± 1.12	30 ± 0.18	66 ± 0.12
**4e**	>100	>100	>100
**5a**	47.5 ± 0.611	52 ± 0.40	30 ± 0.59
**5b**	34.2 ± 0.45	26.34 ± 0.73	21.81 ± 0.67
**5c**	14.4 ± 0.38	20 ± 0.17	15 ± 0.64
**5d**	30.24 ± 0.85	34.83 ± 0.90	27.29 ± 0.74
**5e**	4.46 ± 0.51	3.59 ± 0.42	6.01 ± 0.53
**6a**	>100	72 ± 0.52	60 ± 0.10
**6b**	60 ± 0.93	66.7 ± 0.12	41 ± 0.69
**6c**	41.89 ± 0.61	42 ± 0.48	38 ± 0.94
**6d**	99 ± 0.19	71.2 ± 0.56	86 ± 0.15
**6e**	35.9 ± 0.92	23.45 ± 0.70	40.31 ± 0.99
**5-FU**	8.83 ± 0.09	9.35 ± 0.74	7.51 ± 0.11

^a^ The cytotoxic activity of compounds on the cancer cell lines determined using the sulforhodamine B (SRB) assay. The data are expressed as the mean ± SD of three independent experiments.

**Table 2:** Cytotoxicity of compounds **5c** and **5e** on the normal WI-38 cell line.

Compound	WI-38IC_50_ (µM) ^a^		Selectivity Index ^b^	
		PC-3	MDA-MB-231	HepG2
**4a**	164.25 ± 4.5	-	-	1.50
**4b**	127.18 ± 3.6	-	-	-
**4c**	136.53 ± 4.15	-	-	2.20
**4d**	103.94 ± 3.16	1.96	3.46	1.57
**4e**	94.71 ± 2.5	-	-	-
**5a**	174.11 ± 4.62	3.66	3.34	5.80
**5b**	131.5 ± 4.12	3.84	4.99	6.02
**5c**	91.29 ± 0.47	6.33	4.56	6.08
**5d**	181.15 ± 4.55	5.99	5.20	6.63
**5e**	102.57 ± 0.81	22.99	28.57	17.06
**6a**	97.42 ± 2.88	-	1.35	1.62
**6b**	205.64 ± 4.73	3.42	3.08	5.01
**6c**	89.62 ± 2.75	2.13	2.13	2.35
**6d**	145.80 ± 4.55	1.47	2.04	1.69
**6e**	103.94 ± 3.16	2.89	4.43	2.57
5-FU	7.91 ± 1.80	0.89	0.84	1.05

^a^ The cytotoxic activity of compounds on the cancer cell lines determined using the SRB assay. The data are expressed as the mean ± SD of three independent experiments. ^b^ Selectivity index (SI) = IC_50_ on WI-38/IC_50_ on cancer cells

**Table 3:** Results of cell cycle analysis in MDA-MB-231cells expressed by (%) of cells in each phase after treatment with compounds **5c** and **5e**.

Compound	%G0-G1	%S	%G2-M	%Pre-G1
**5c**	45.27	33.16	21.57	16.32
**5e**	42.36	32.48	25.16	19.47
**Control**	60.41	35.21	4.38	1.87

**Table 4:** Percent of cell death induced by compounds **5c** and **5e** on MDA-MB-231cells.

Compound	Apoptosis		Necrosis	Total
	Early	Late		
**5c**	5.07	8.67	2.58	16.32
**5e**	6.18	10.91	2.38	19.47
**Control**	1.09	0.31	0.47	1.87

**Table 5:** The docking results of compounds **5c** and **5e** into the active site of survivin enzyme.

Compound	Docking Score (kcal/mol)	Interacting Residues	Distance (A°)
**5c**	−6.7164	Thr97 Leu98	3.24 3.39
**5e**	−7.6630	Thr97 Leu98	3.01 2.76
**LLP9**	−8.0721	Thr97 Leu98	3.26 2.71
**4a**	−4.9213	-	-

## Supplementary Materials

The Supplementary Materials are available online, Figures S1–S36 contain the NMR charts for the synthesized compounds, Figures S37–S39 contain the cell cycle analysis for control and compounds **5c** and **5e**, Figures S40–S41 contain western blotting analysis for **5c** and **5e**.
