# Supplementary material for: Discovery of Novel 3-Cyanopyridines as Survivin Modulators and Apoptosis Inducers
Source: Molecules. 2020 Oct 22;25(21):4892. doi: 10.3390/molecules25214892 (PMC7660103; doi:10.3390/molecules25214892)
Supplement: Supplementary file 1 [file molecules-25-04892-s001.pdf]

Synthesis and discovery of new 3-cyanopyridine derivatives as anti-tumor and apoptosis inducers targeting survivin

Contents

- **Figure S1**. ^{1}HNMR spectrum of **3a**
- **Figure S2.** ^{13}CNMR spectrum of **3a**
- **Figure S3.** ^{1}HNMR spectrum of **3b**
- **Figure S4.** ^{13}CNMR spectrum of **3b**
- **Figure S5.** ^{1}HNMR spectrum of **3c**
- **Figure S6.** ^{13}CNMR spectrum of **3c**
- **Figure S7.** ^{1}HNMR spectrum of **3d**
- **Figure S8.** ^{13}CNMR spectrum of **3d**
- **Figure S9.** ^{1}HNMR spectrum of **3e**
- **Figure S10.** ^{13}CNMR spectrum of **3e**
- **Figure S11.** ^{1}HNMR spectrum of **4b**
- **Figure S12.** ^{13}CNMR spectrum of **4b**
- **Figure S13.** ^{1}HNMR spectrum of **4c**
- **Figure S14.** ^{13}CNMR spectrum of **4c**
- **Figure S15.** ^{1}HNMR spectrum of **4d**
- **Figure S16.** ^{13}CNMR spectrum of **4d**
- **Figure S17.** ^{1}HNMR spectrum of **4e**
- **Figure S18.** ^{13}CNMR spectrum of **4e**
- **Figure S19.** ^{1}HNMR spectrum of **5a**
- **Figure S20.** ^{13}CNMR spectrum of **5a**
- **Figure S21.** ^{1}HNMR spectrum of **5b**
- **Figure S22.** ^{13}CNMR spectrum of **5b**
- **Figure S23.** ^{1}HNMR spectrum of **5c**
- **Figure S24.** ^{13}CNMR spectrum of **5c**
- **Figure S25.** ^{1}HNMR spectrum of **5d**
- **Figure S26.** ^{13}CNMR spectrum of **5d**
- **Figure S27.** ^{1}HNMR spectrum of **5e**
- **Figure S28.** ^{13}CNMR spectrum of **5e**
- **Figure S29.** ^{1}HNMR spectrum of **6b**
- **Figure S30.** ^{13}CNMR spectrum of **6b**
- **Figure S31.** ^{1}HNMR spectrum of **6c**
- **Figure S32.** ^{13}CNMR spectrum of **6c**
- **Figure S33.** ^{1}HNMR spectrum of **6d**
- **Figure S34.** ^{13}CNMR spectrum of **6d**
- **Figure S35.** ^{1}HNMR spectrum of **6e**
- **Figure S36.** ^{13}CNMR spectrum of **6e**
- **Figure S37.** Cell cycle analysis and Annexin V-FITC apoptosis assessment for control MDA-BA 231
- **Figure S38.** Cell cycle analysis and Annexin V-FITC apoptosis assessment for **5c**

- **Figure S39.** Cell cycle analysis and Annexin V-FITC apoptosis assessment for **5e**
- **Figure S40.** Western blotting assay analysis for **5c**
- **Figure S41.** Western blotting assay analysis for **5e**

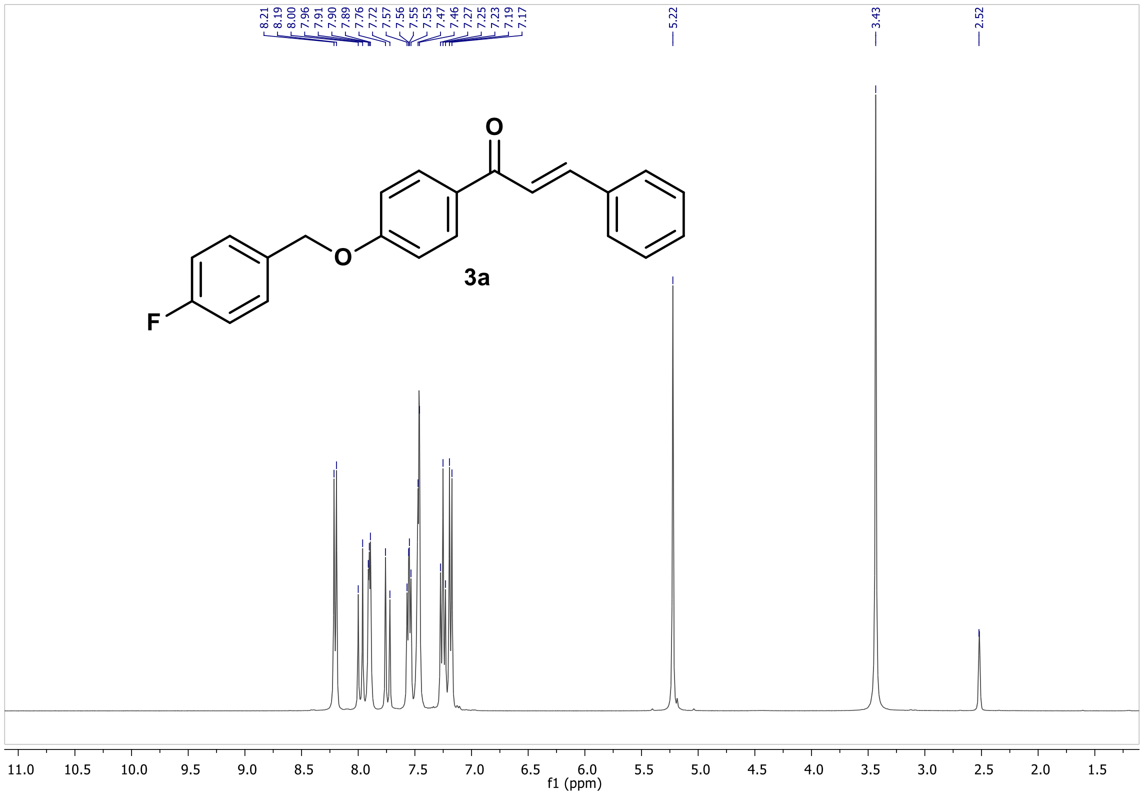

Figure S1. ^{1}HNMR spectrum of **3a**

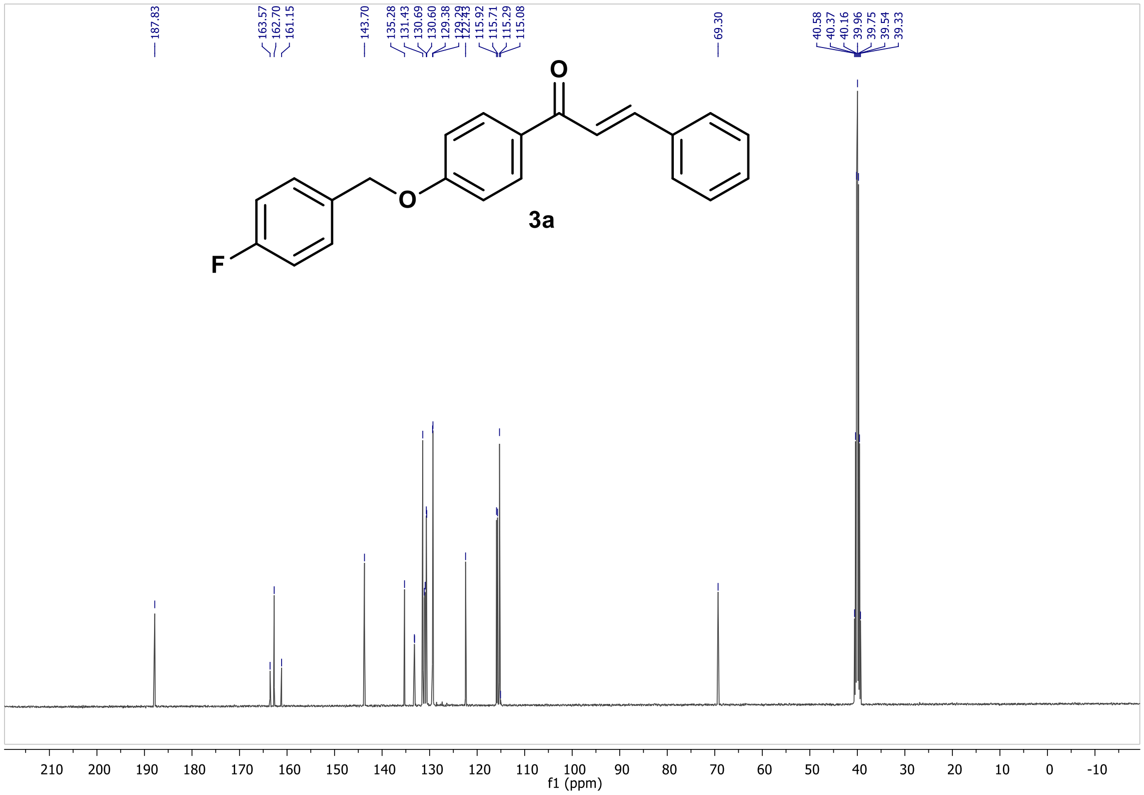

Figure S2. ^{13}CNMR spectrum of **3a**

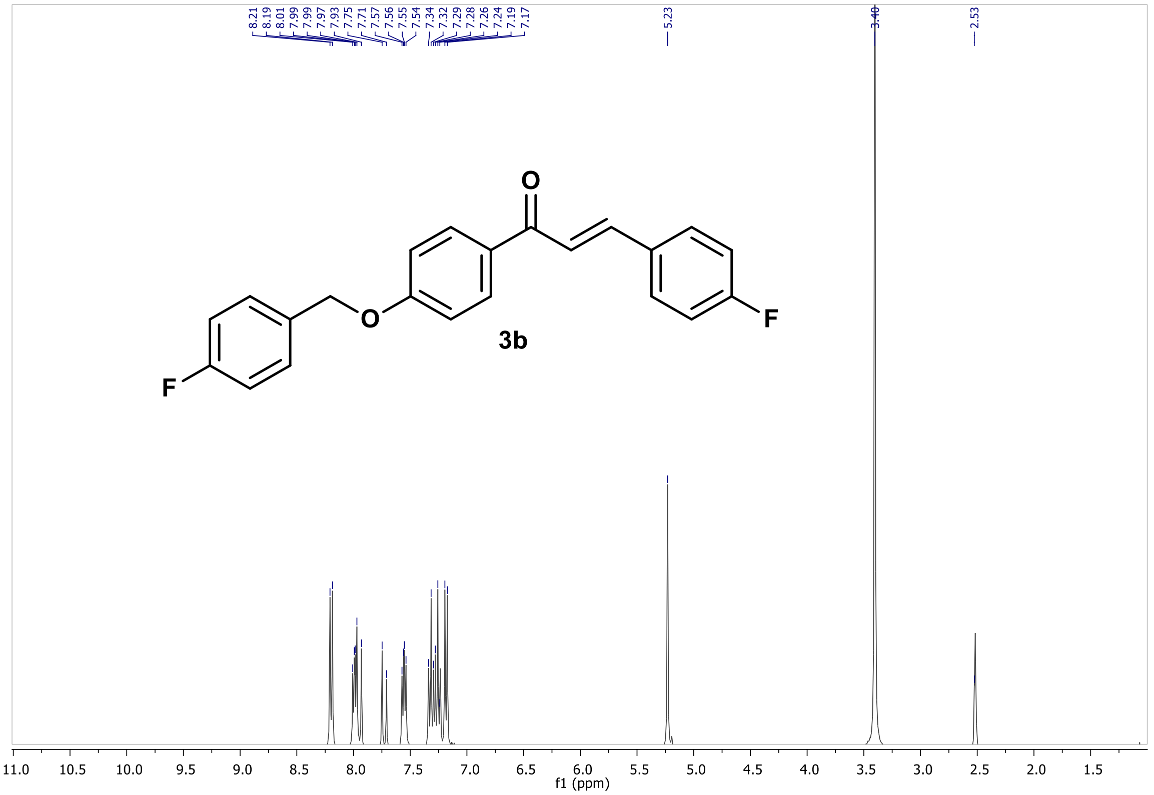

Figure S3. ^{1}HNMR spectrum of **3b**

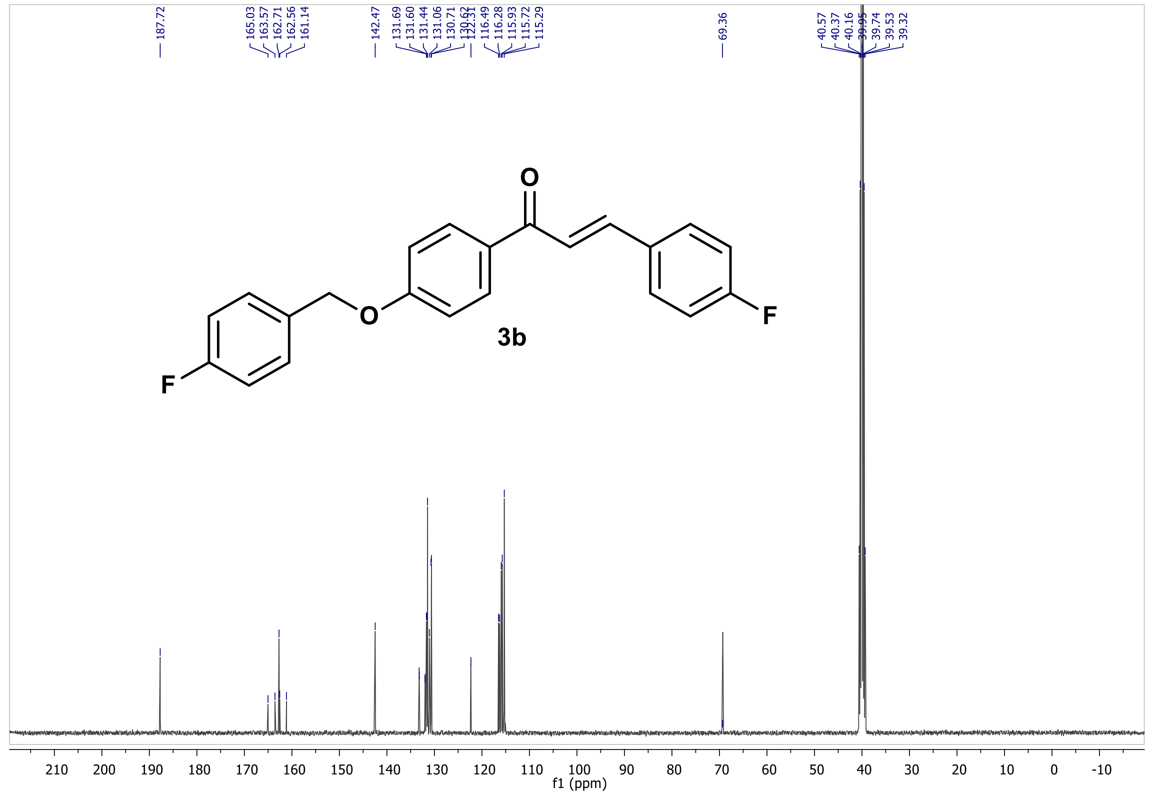

Figure S4. ^{13}CNMR spectrum of **3b**

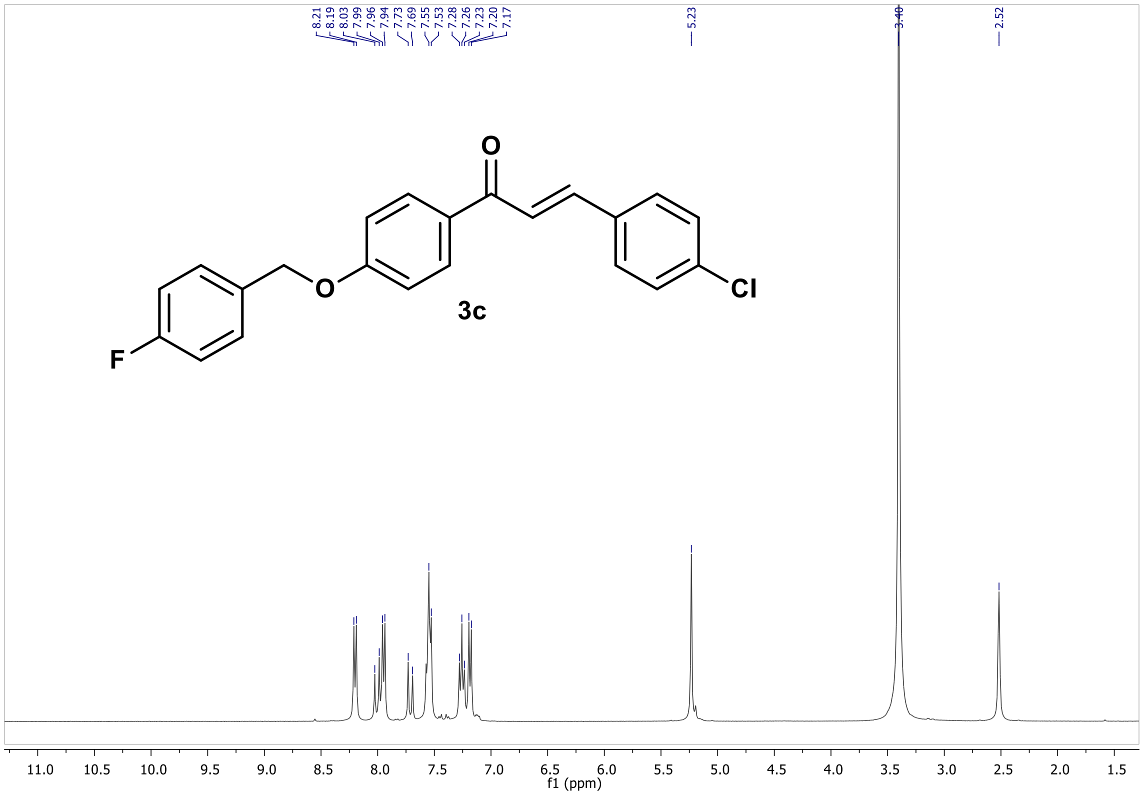

Figure S5. ^{1}HNMR spectrum of **3c**

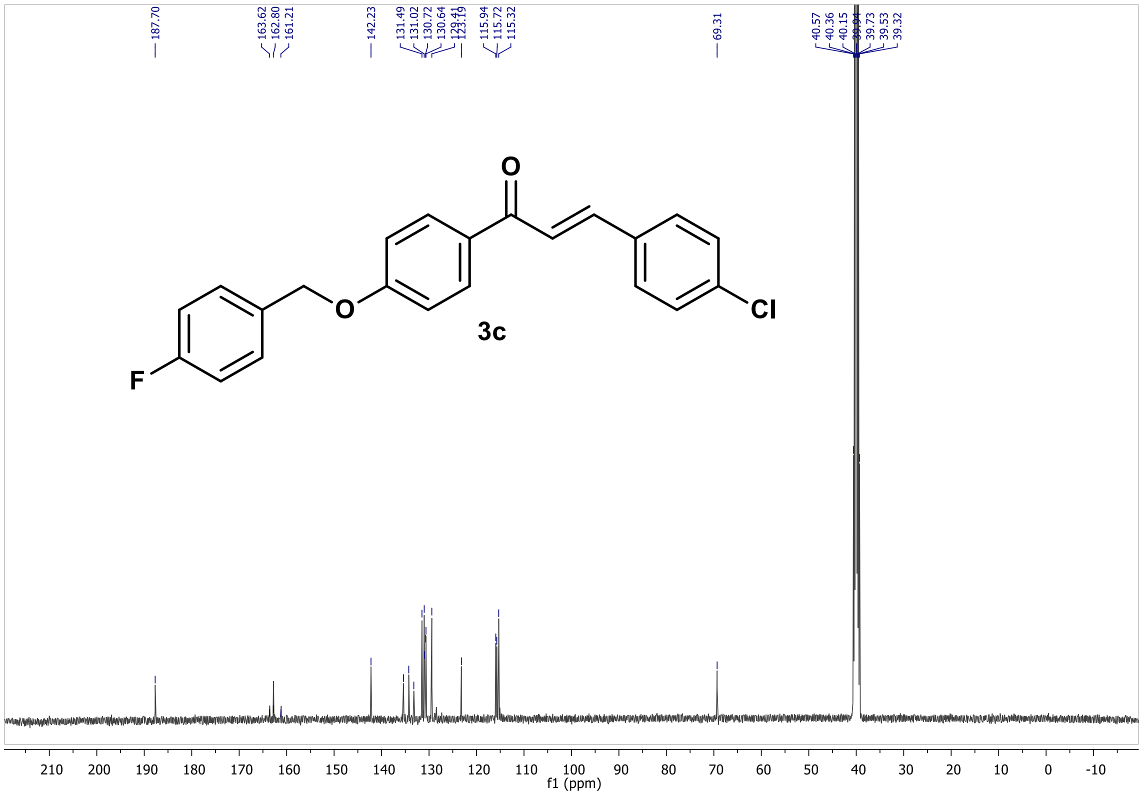

Figure S6. ^{13}CNMR spectrum of **3c**

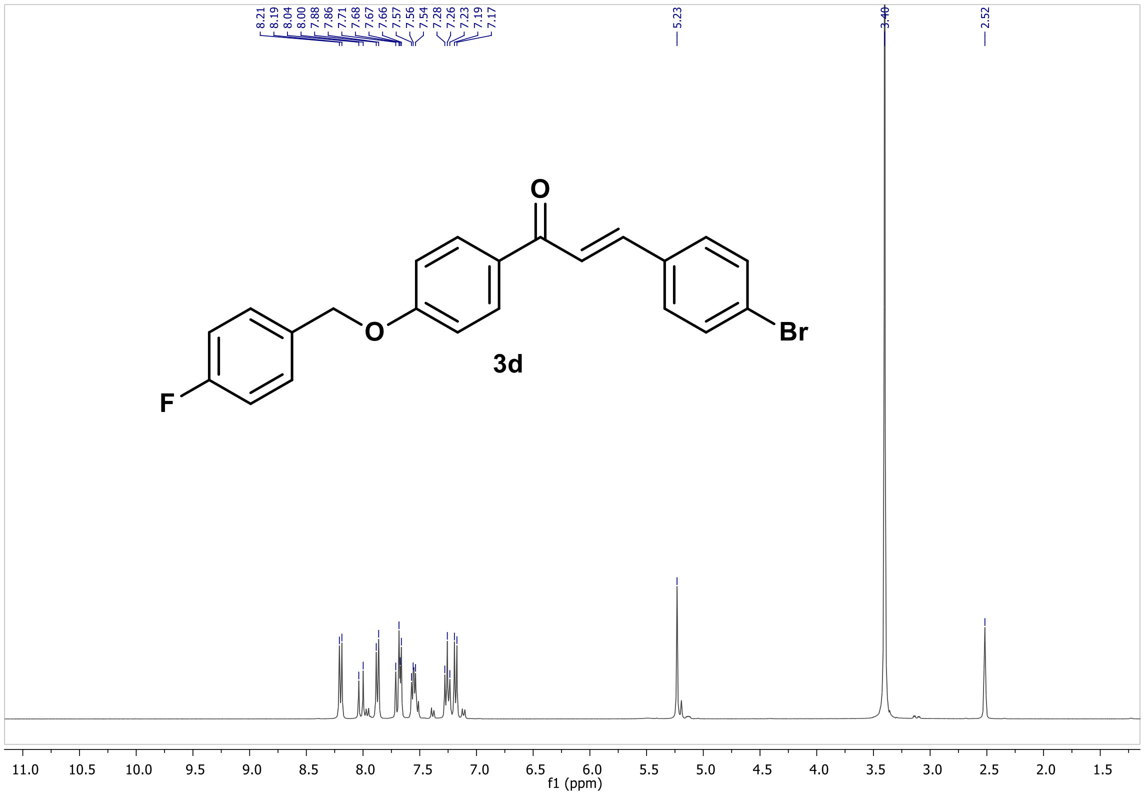

Figure S7. ^{1}HNMR spectrum of **3d**

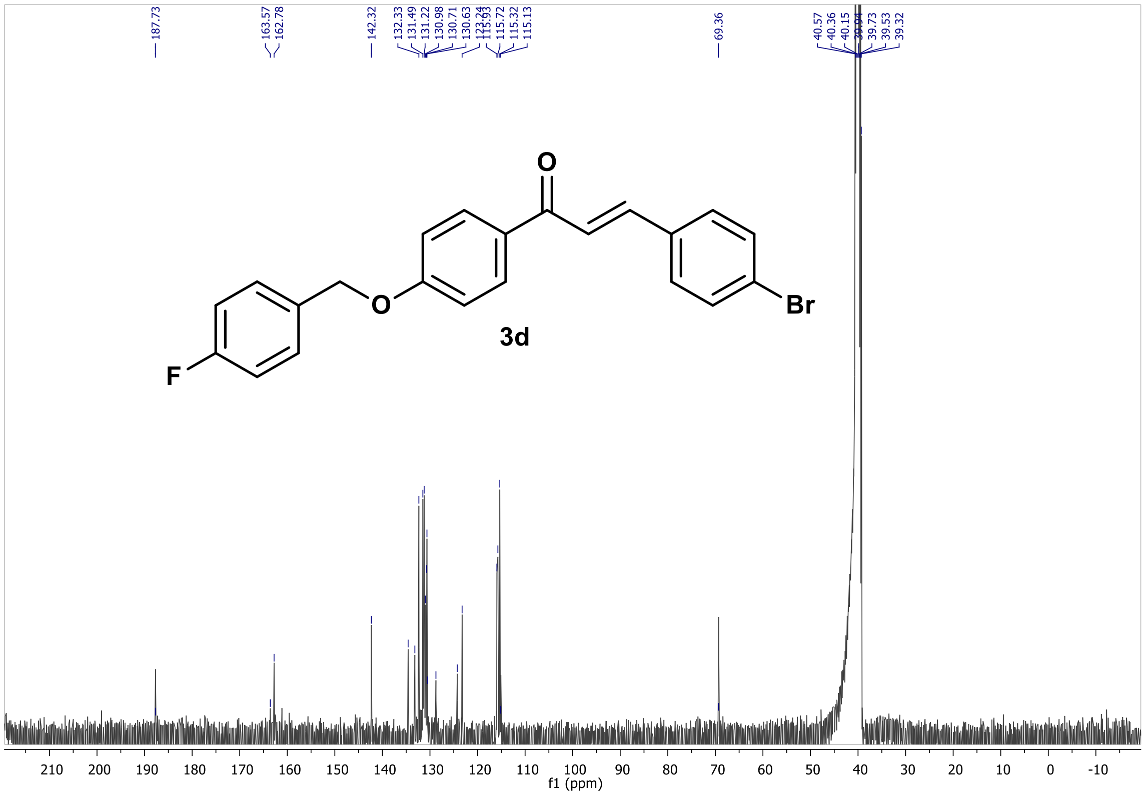

Figure S8. ^{13}CNMR spectrum of **3d**

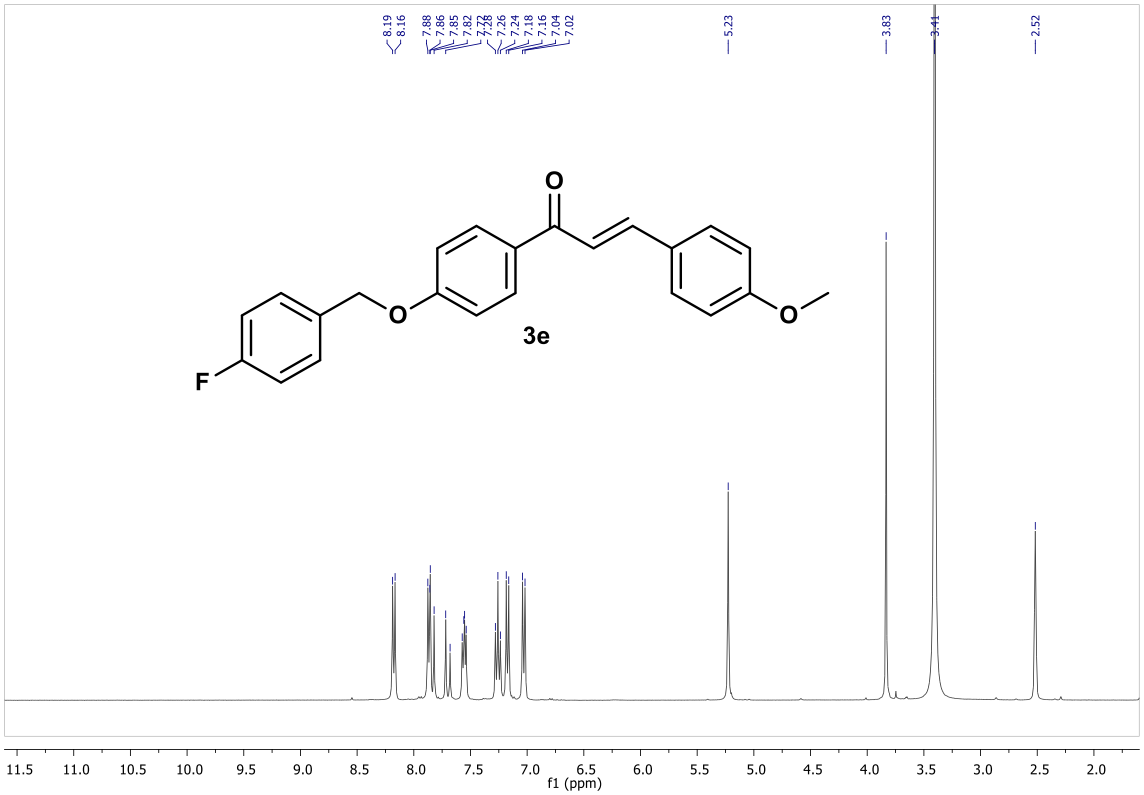

Figure S9. ^{1}HNMR spectrum of **3e**

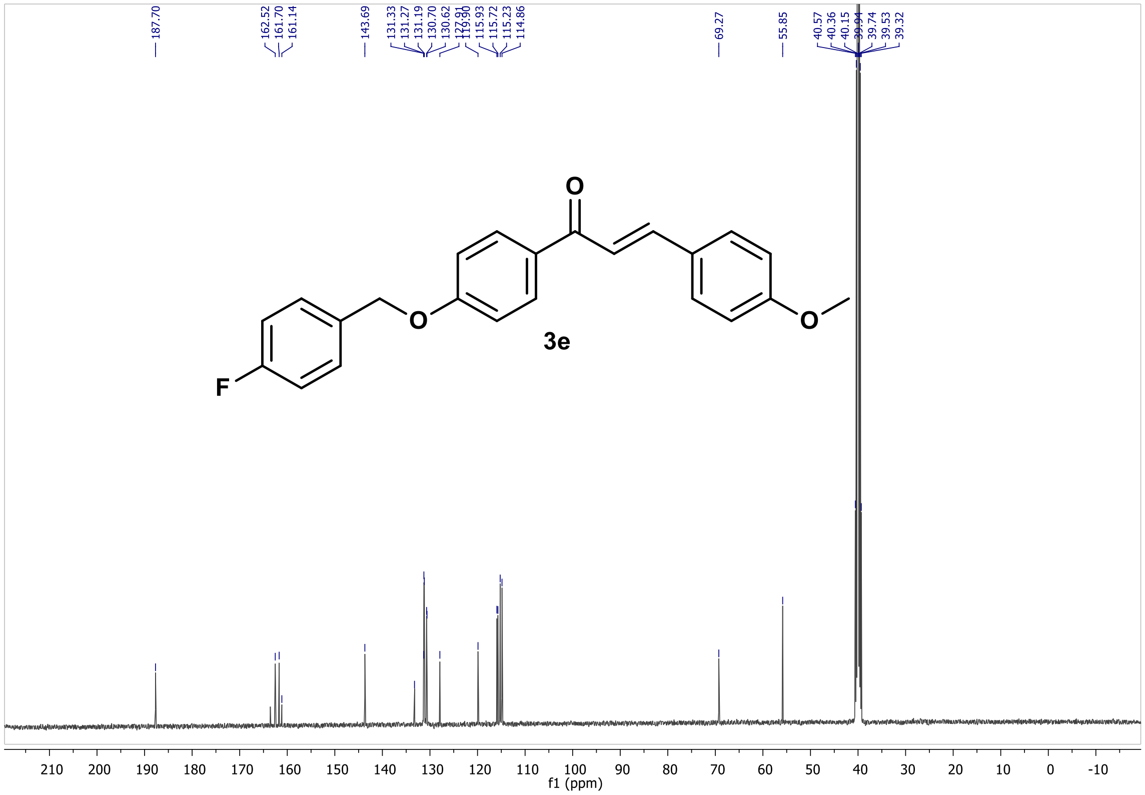

Figure S10. ^{13}CNMR spectrum of **3e**

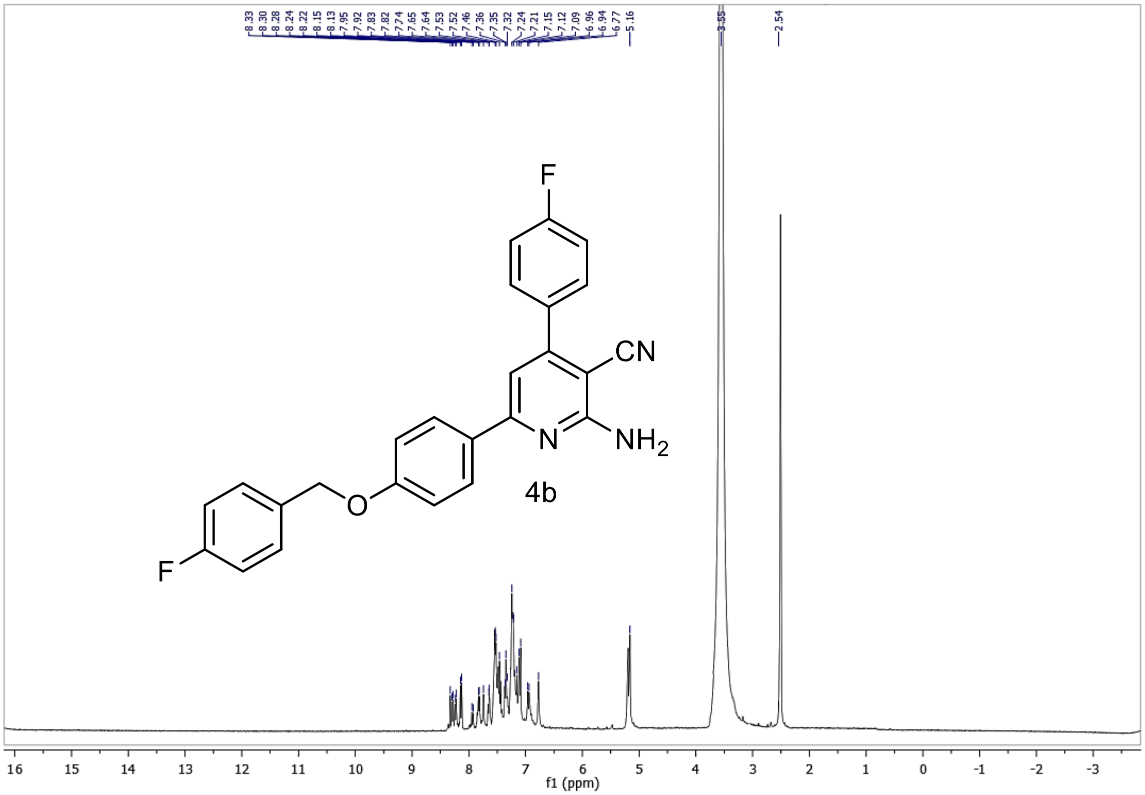

Figure S11. ^{1}HNMR spectrum of **4b**

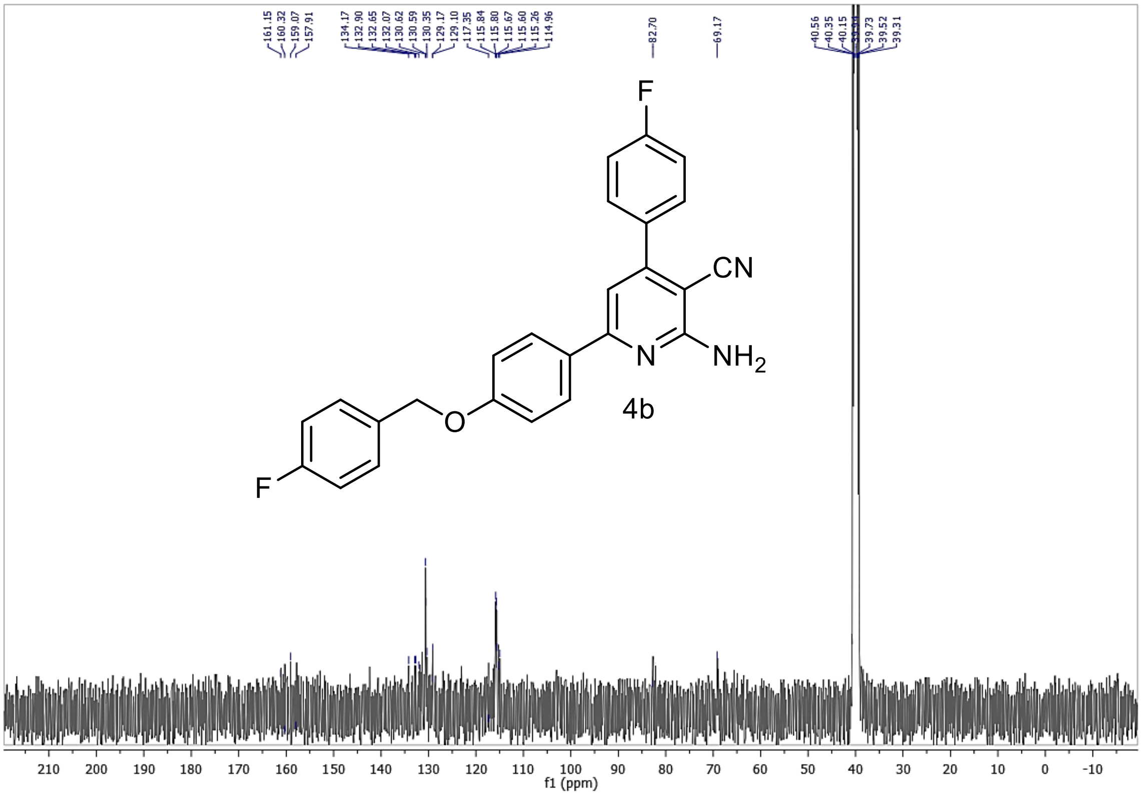

Figure S12. ^{13}CNMR spectrum of **4b**

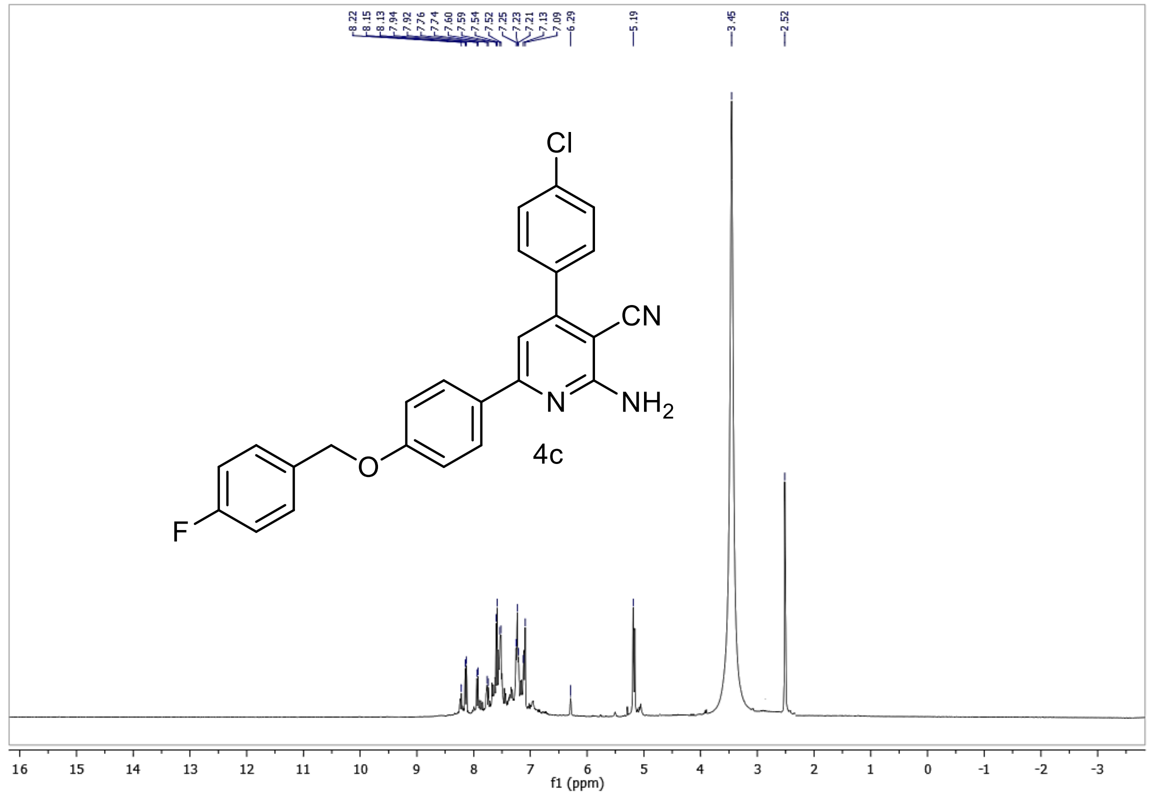

Figure S13. ^{1}HNMR spectrum of **4c**

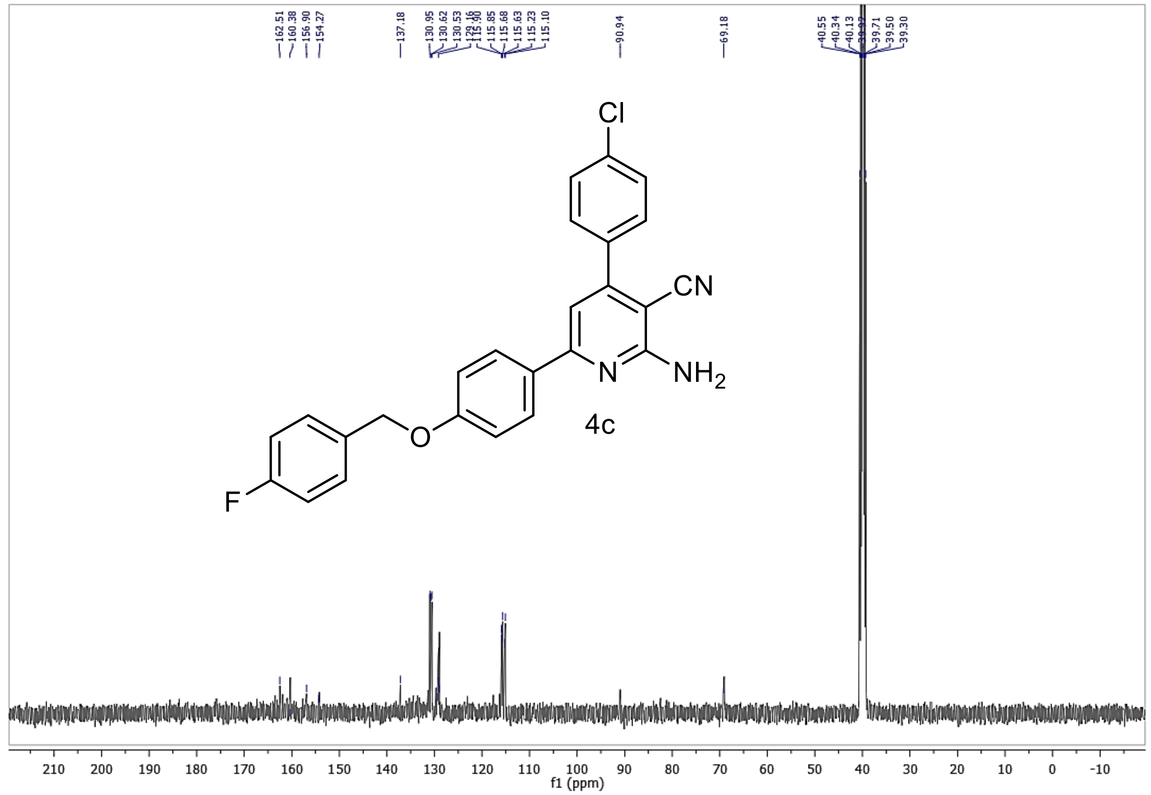

Figure S14. ^{13}CNMR spectrum of **4b**

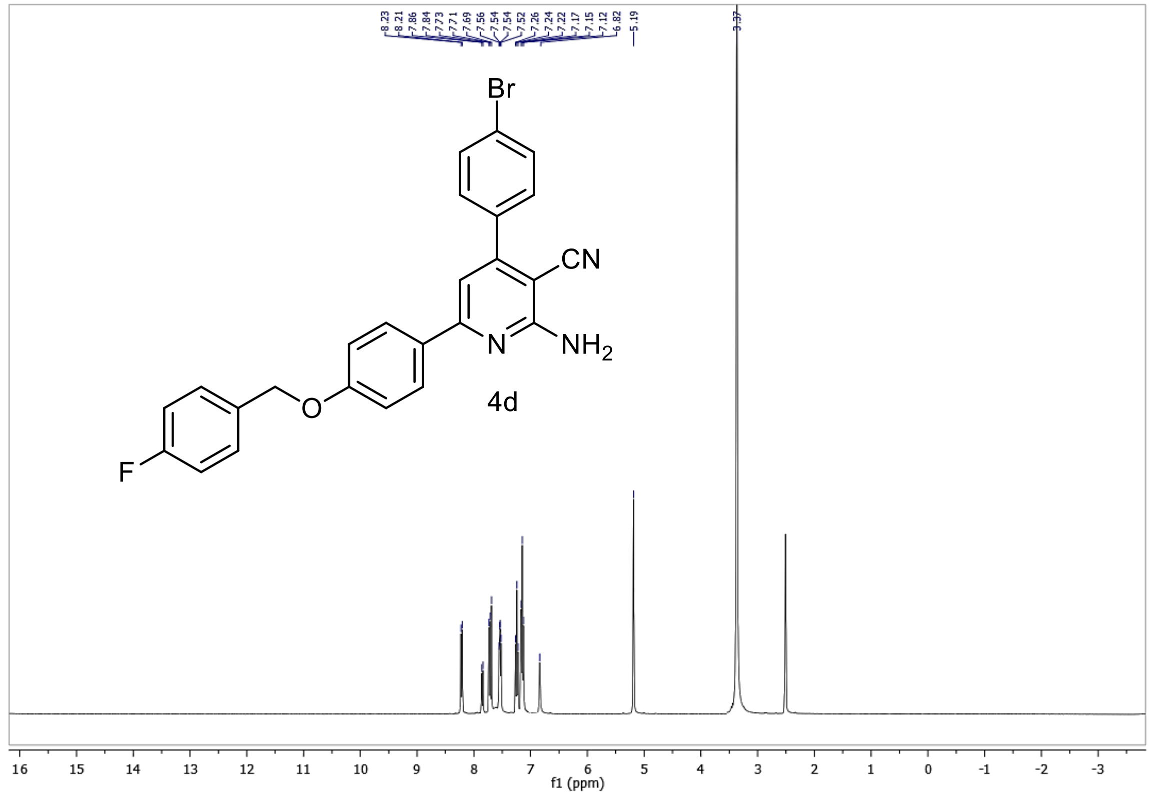

Figure S15. ^{1}HNMR spectrum of **4d**

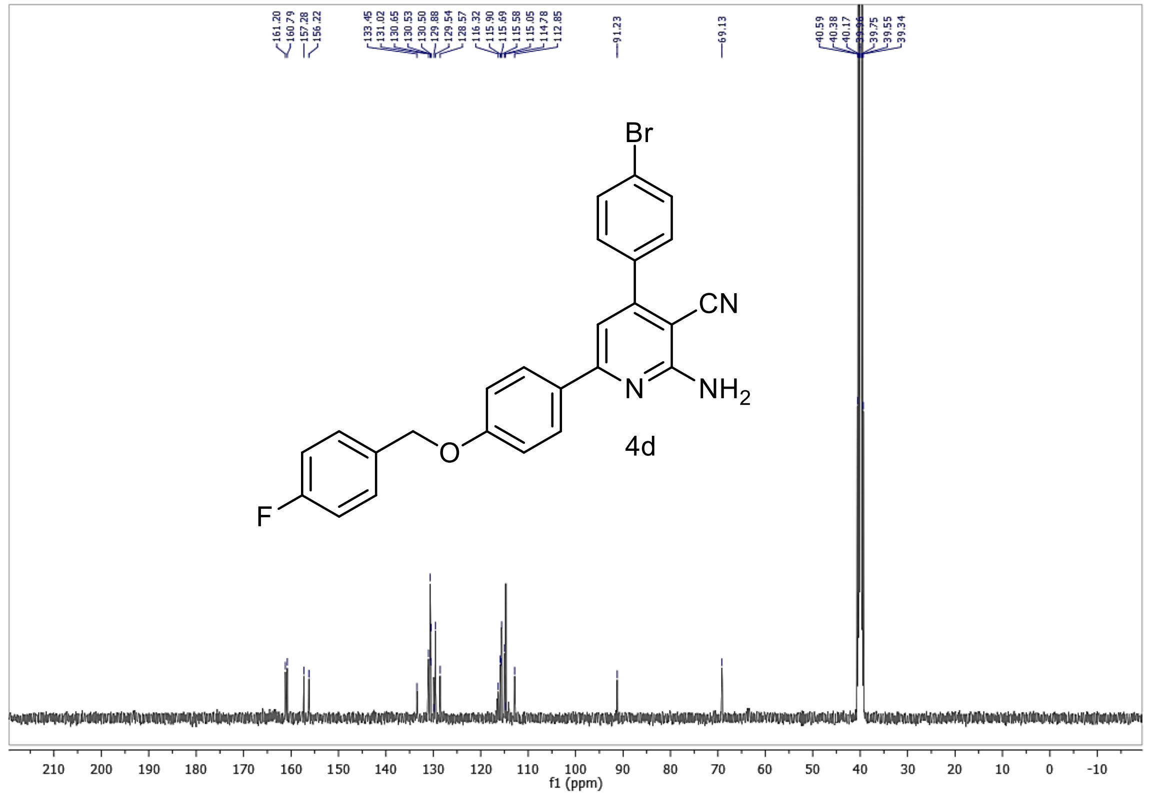

Figure S16. ^{13}CNMR spectrum of **4d**

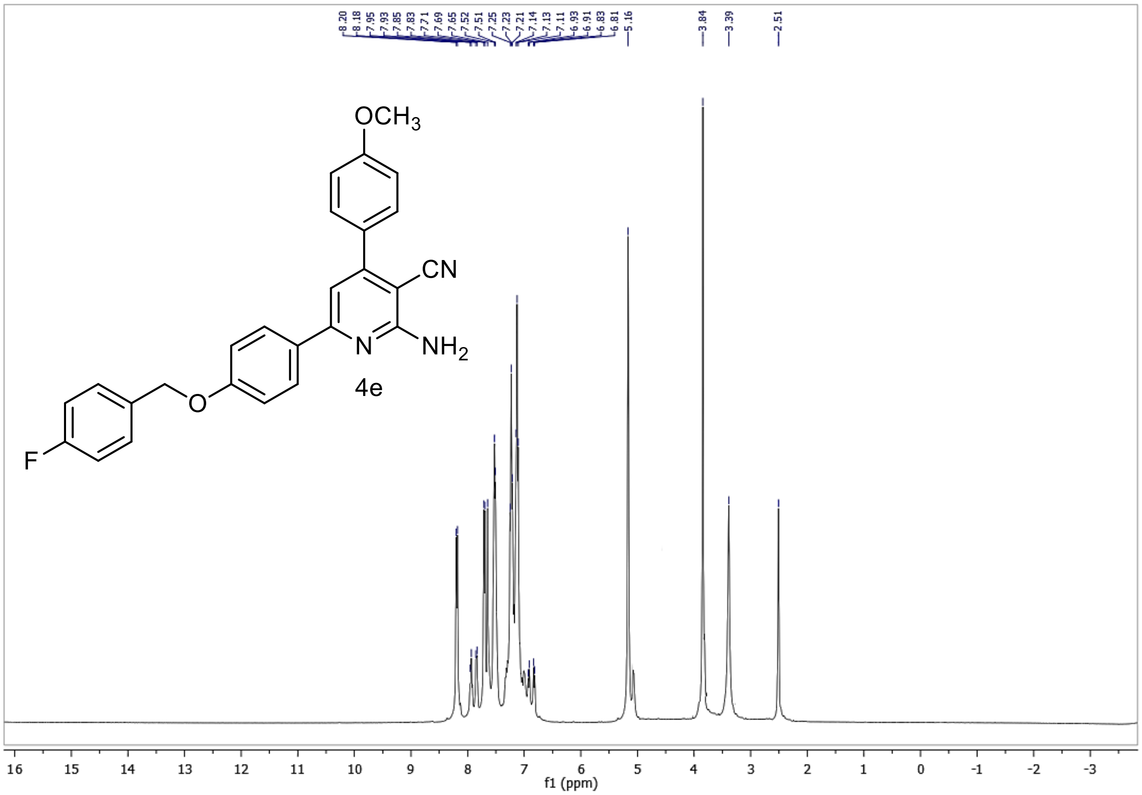

Figure S17. ^{1}HNMR spectrum of **4e**

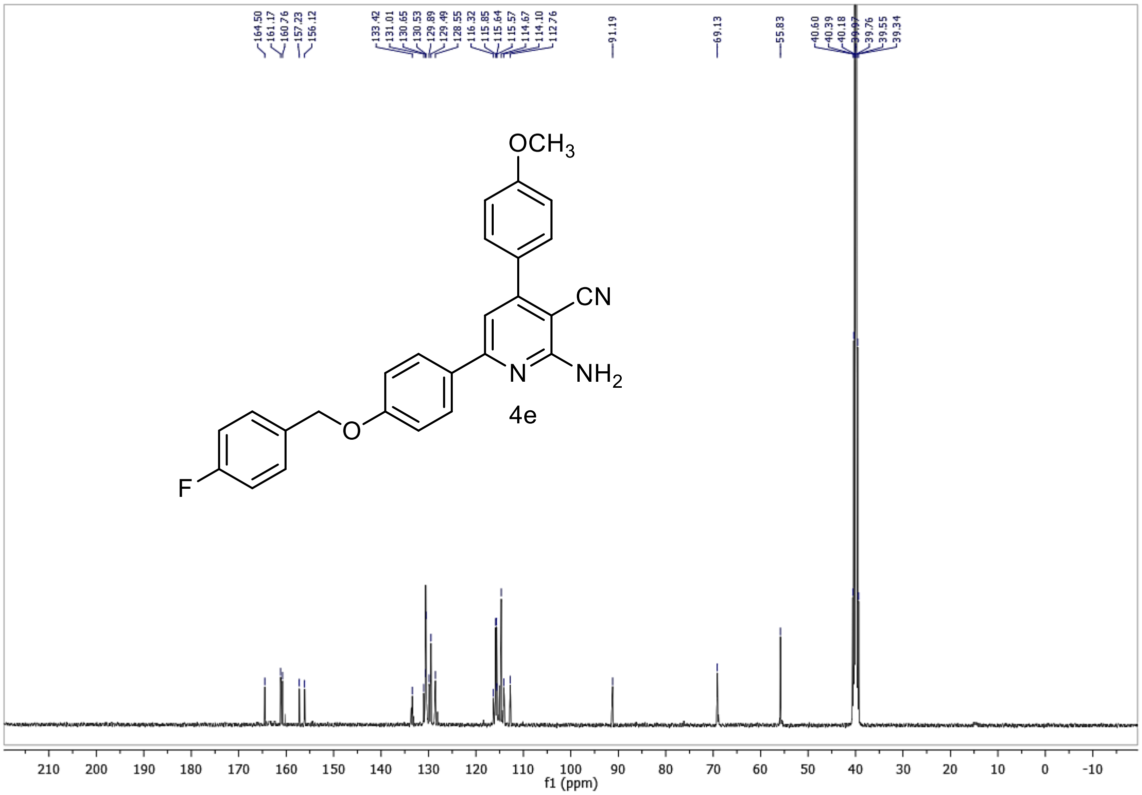

Figure S18. ^{13}CNMR spectrum of **4e**

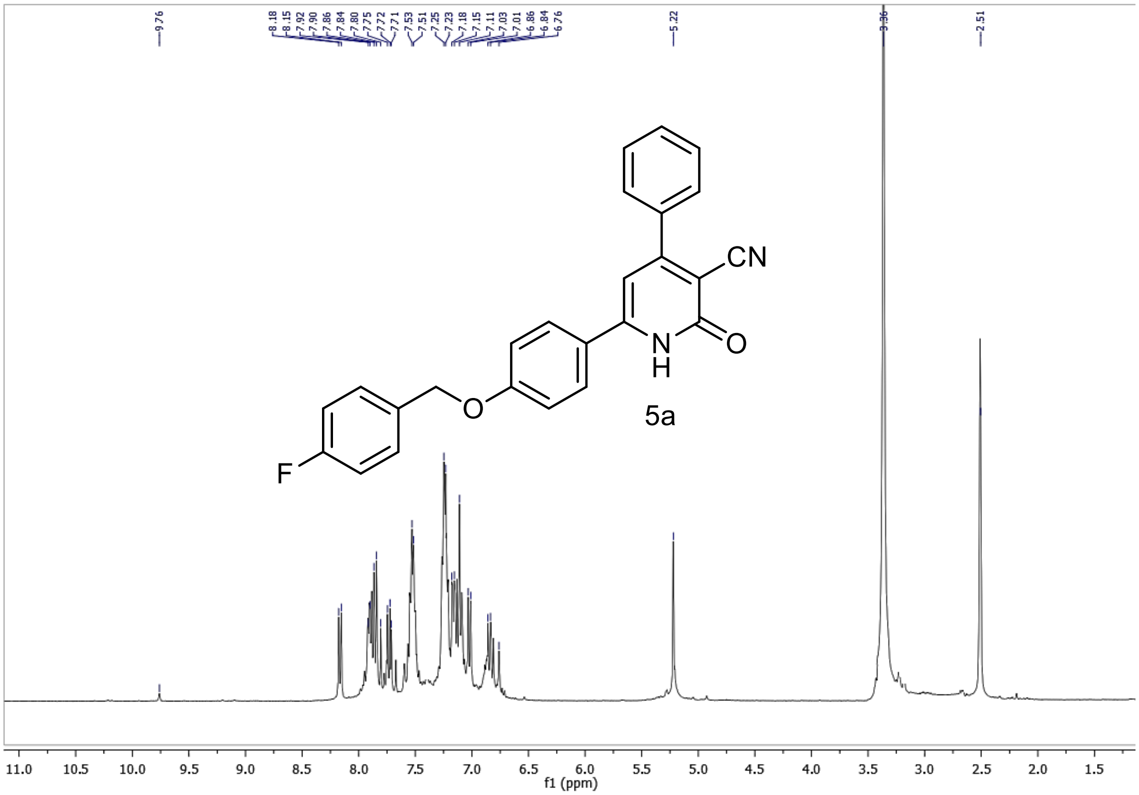

Figure S19. ^{1}HNMR spectrum of **5a**

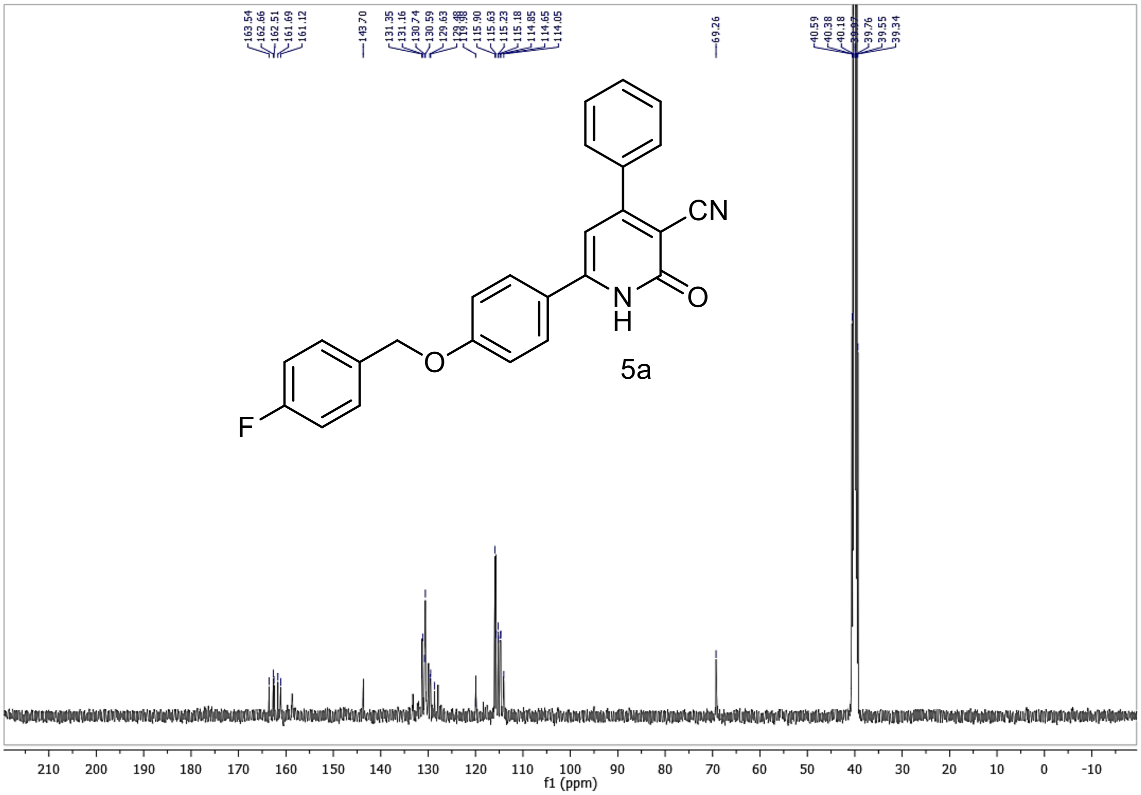

Figure S20. ^{13}CNMR spectrum of **5a**

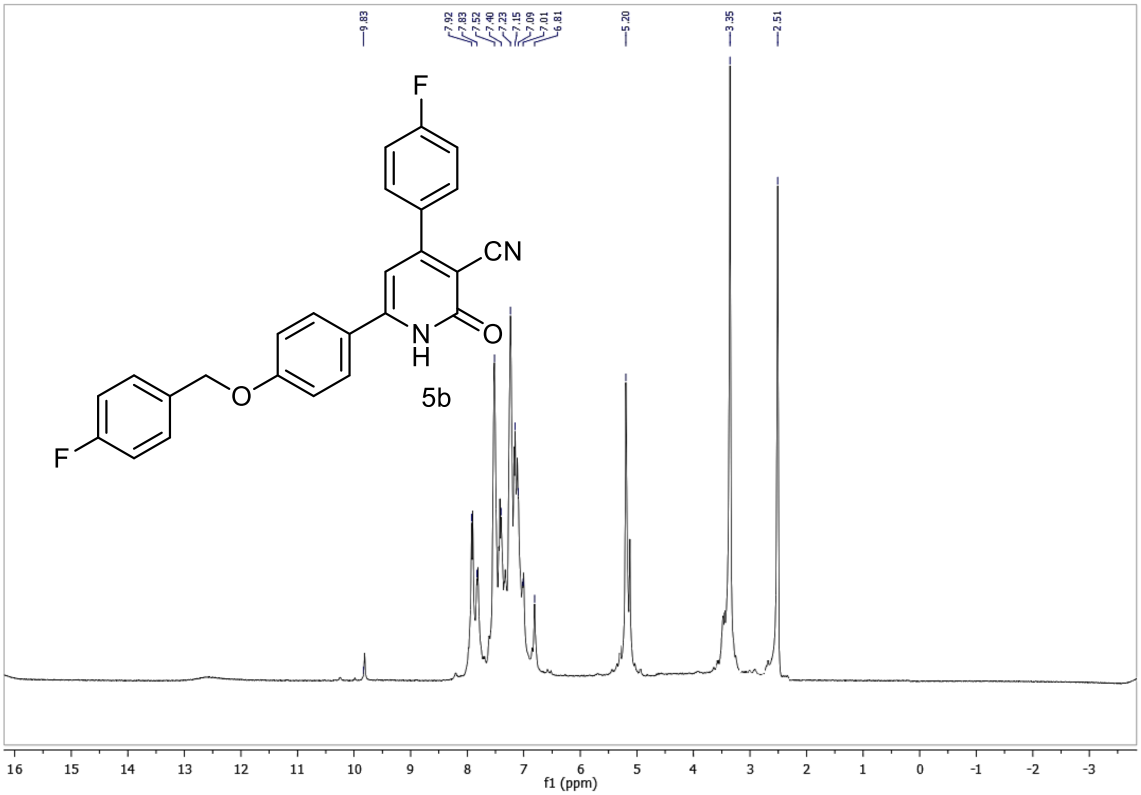

Figure S21. ^{1}HNMR spectrum of **5b**

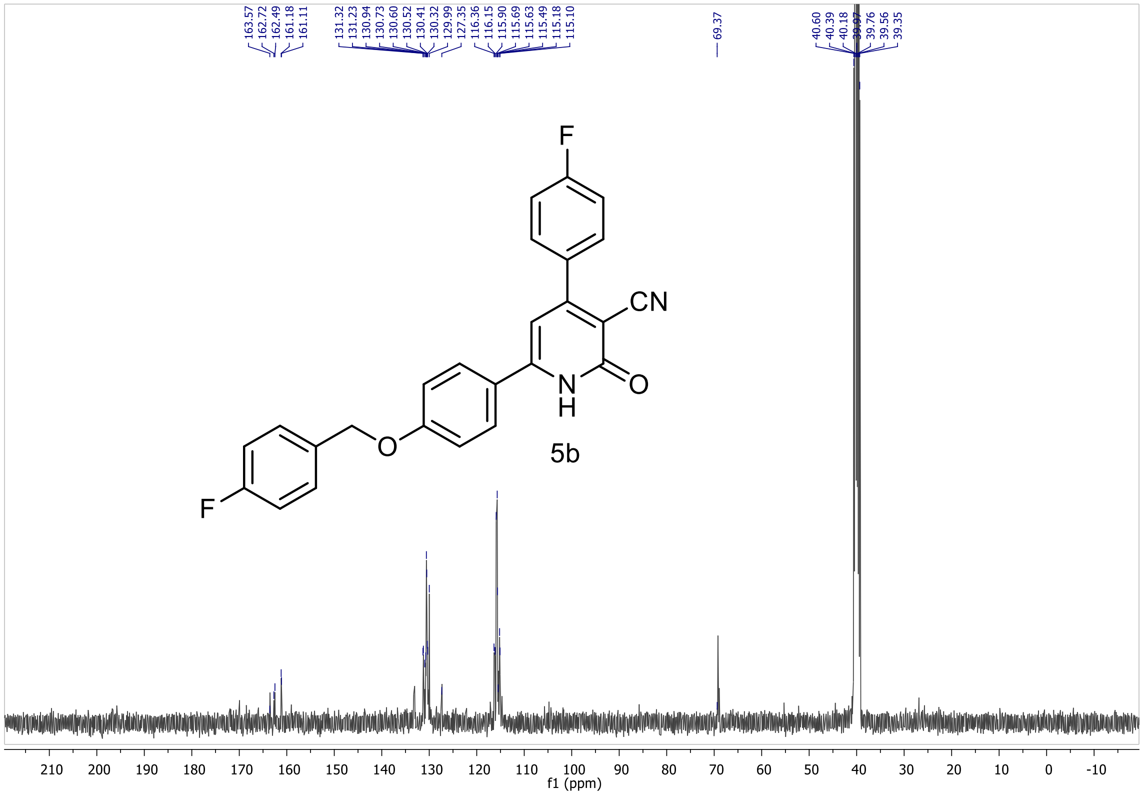

Figure S22. ^{13}CNMR spectrum of **5b**

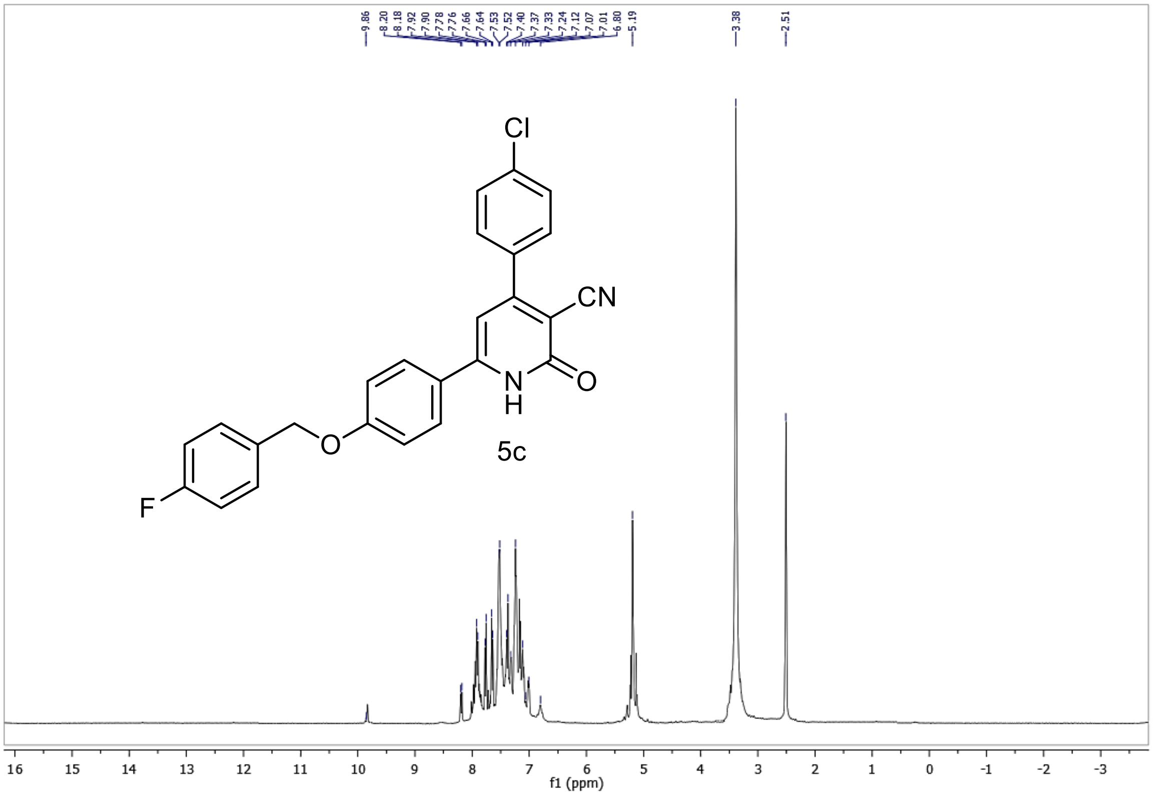

Figure S23. ^{1}HNMR spectrum of **5c**

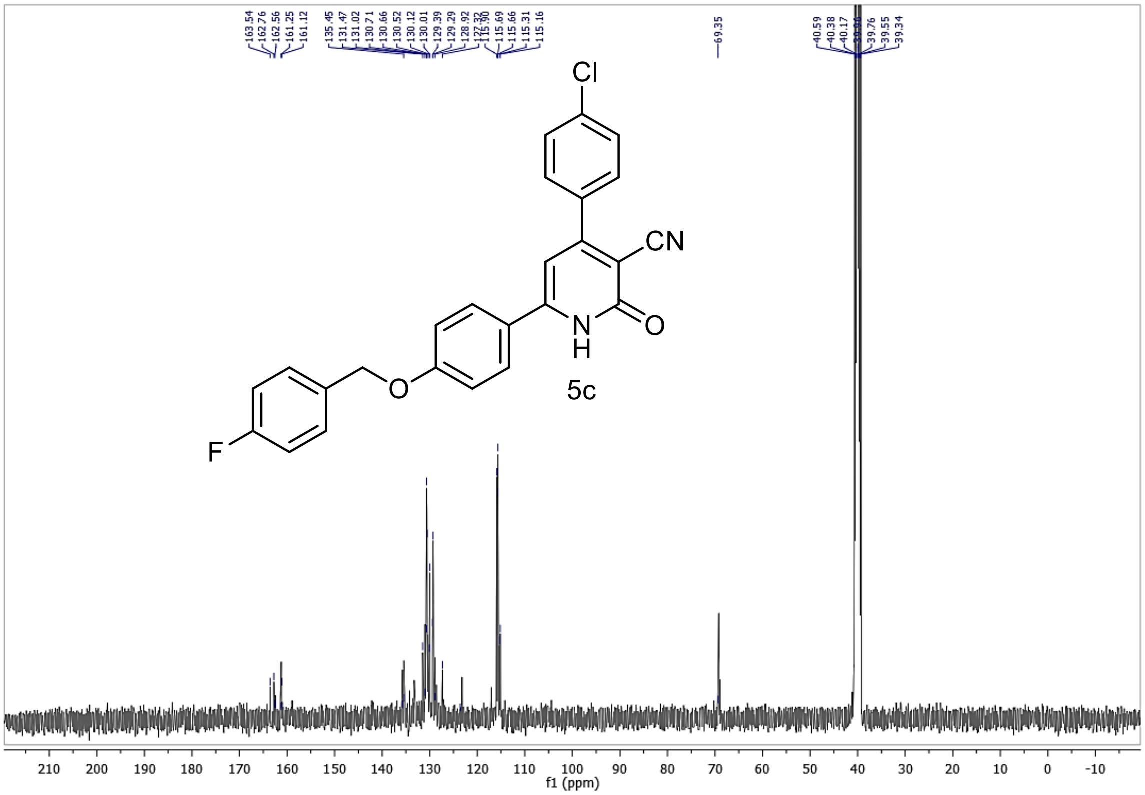

Figure S24. ^{13}CNMR spectrum of **5c**

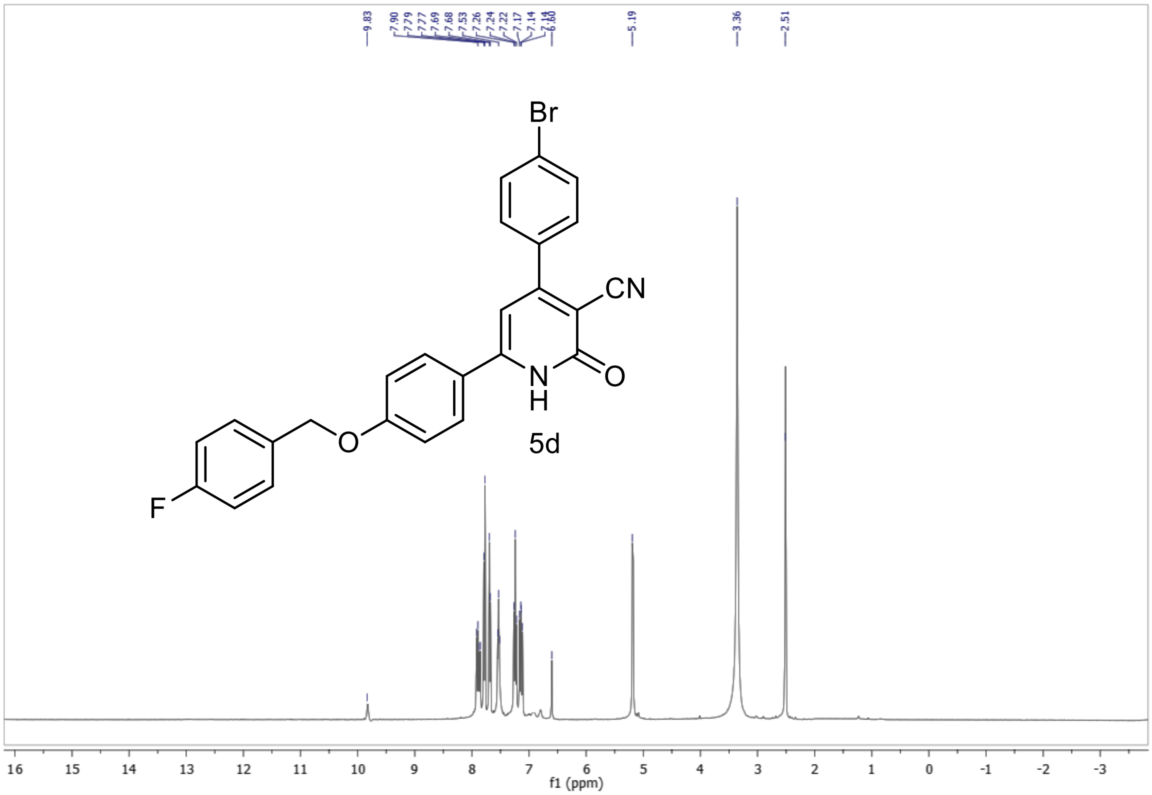

Figure S25. ^{1}HNMR spectrum of **5d**

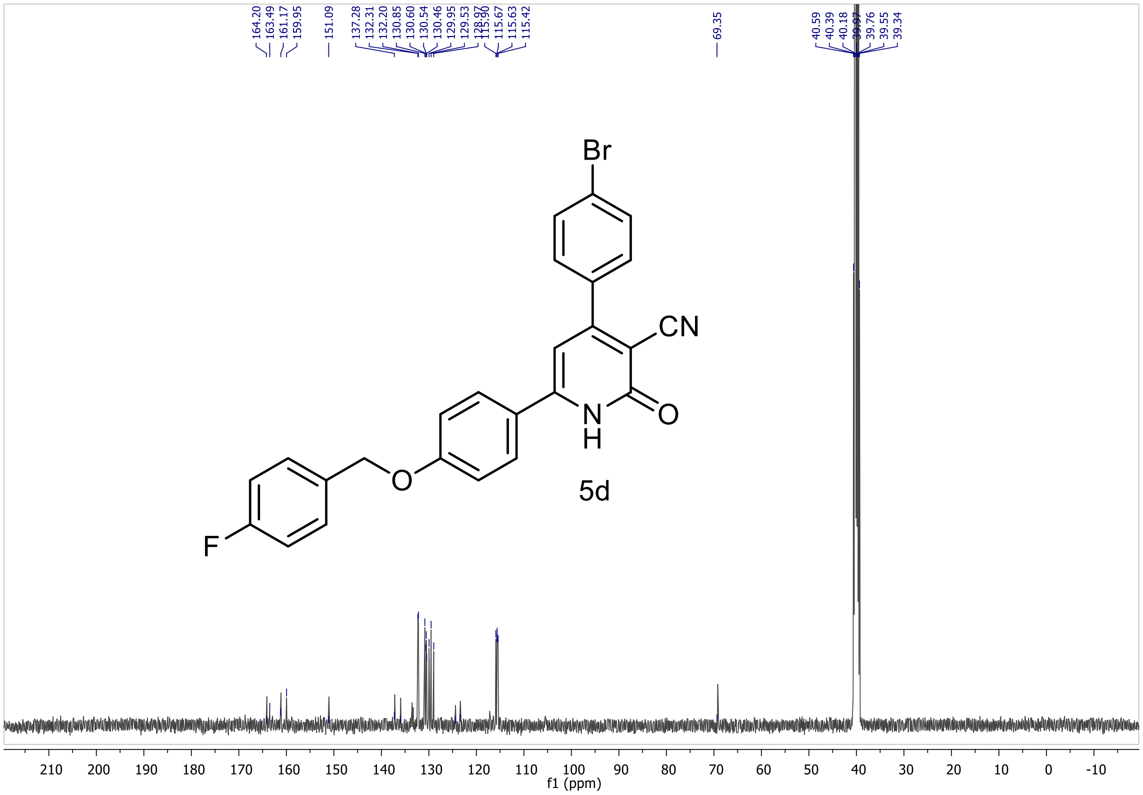

Figure S26. ^{13}CNMR spectrum of **5d**

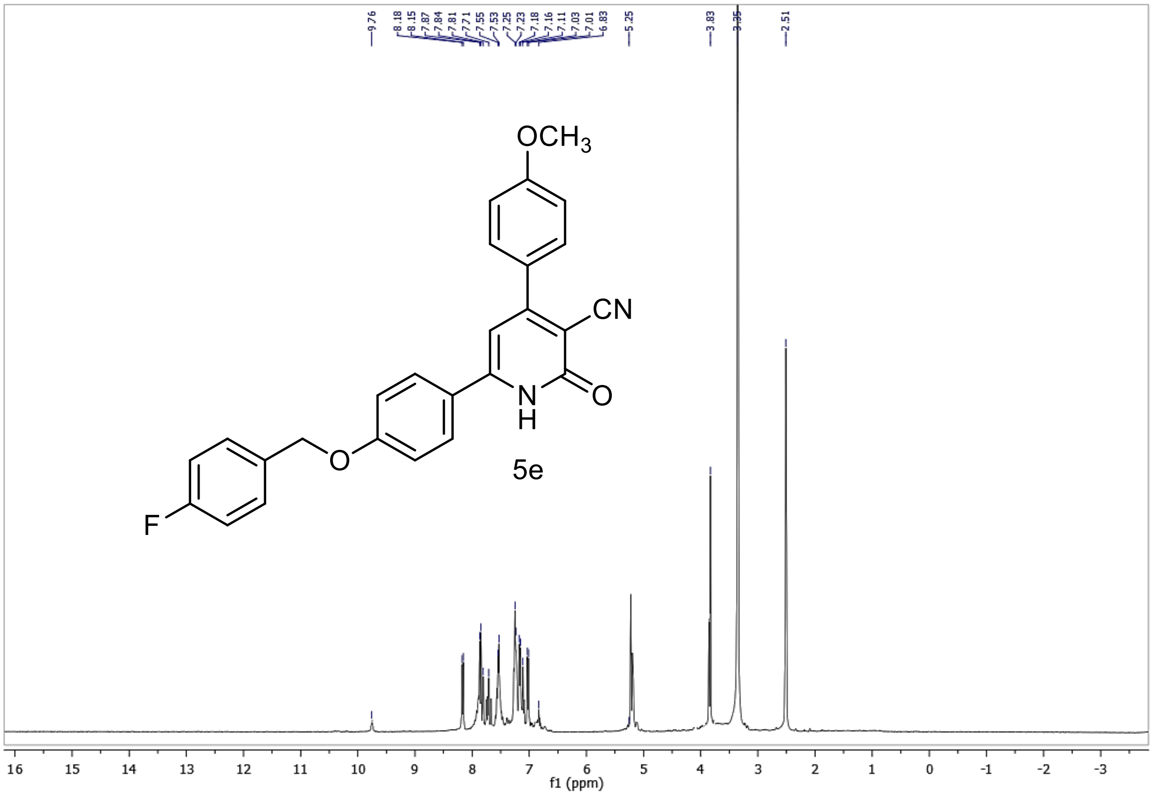

Figure S27. ^{1}HNMR spectrum of **5e**

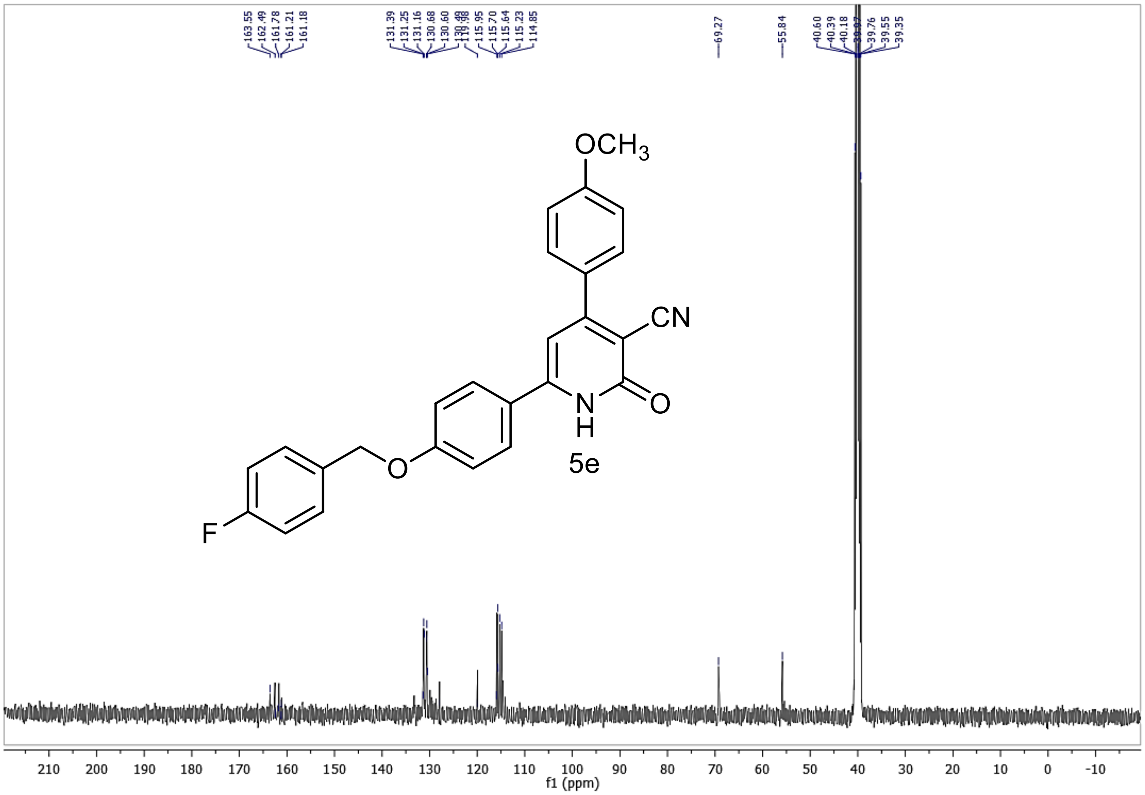

Figure S28. ^{13}CNMR spectrum of **5e**

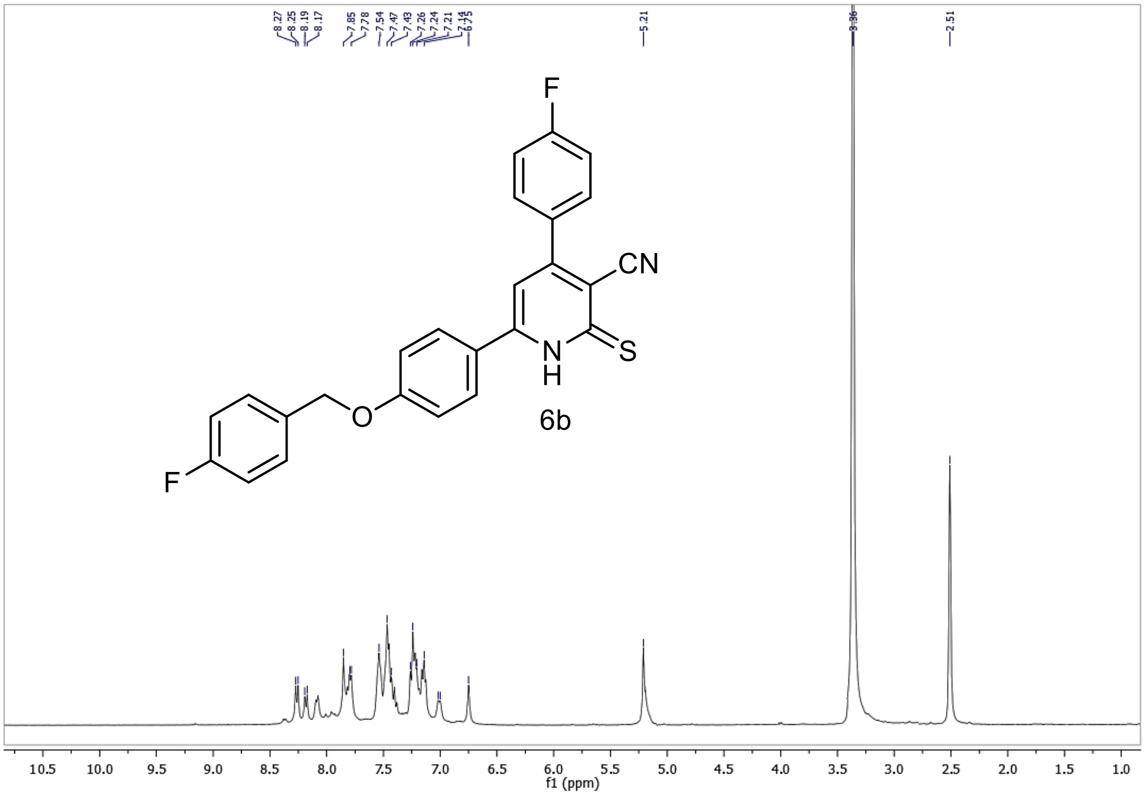

Figure S29. ^{1}HNMR spectrum of **6b**

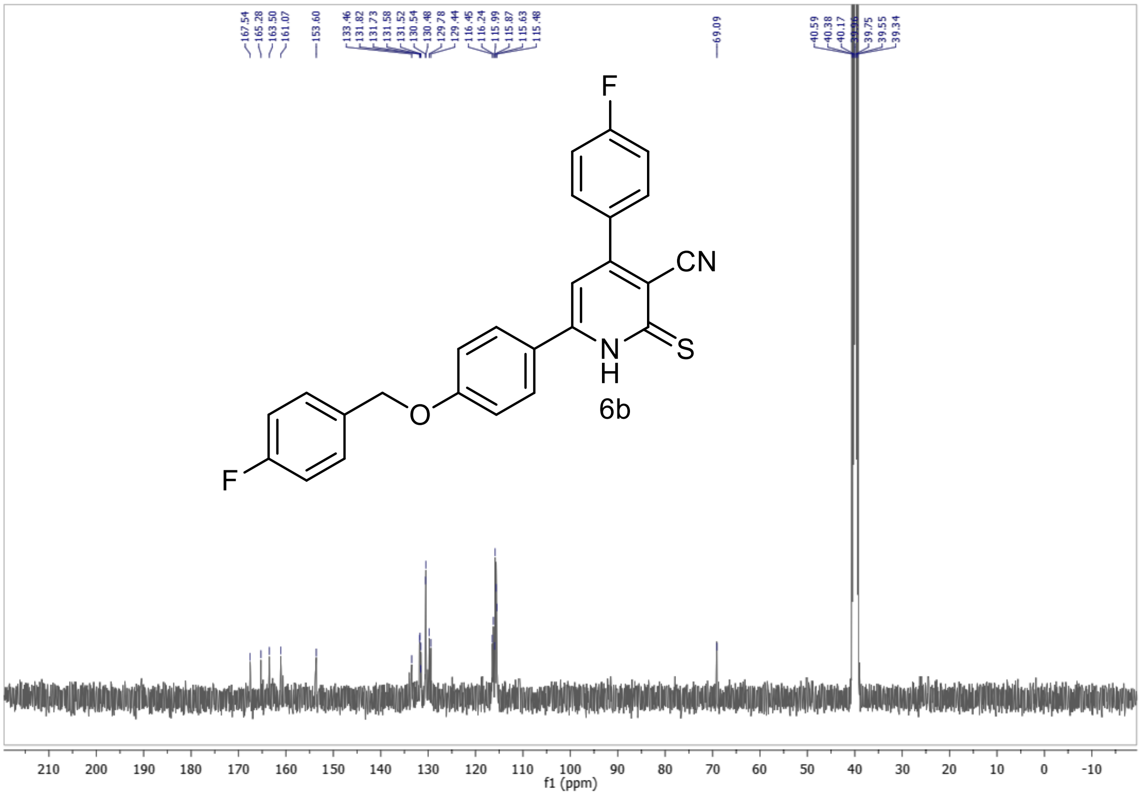

Figure S30. ^{13}CNMR spectrum of **6b**

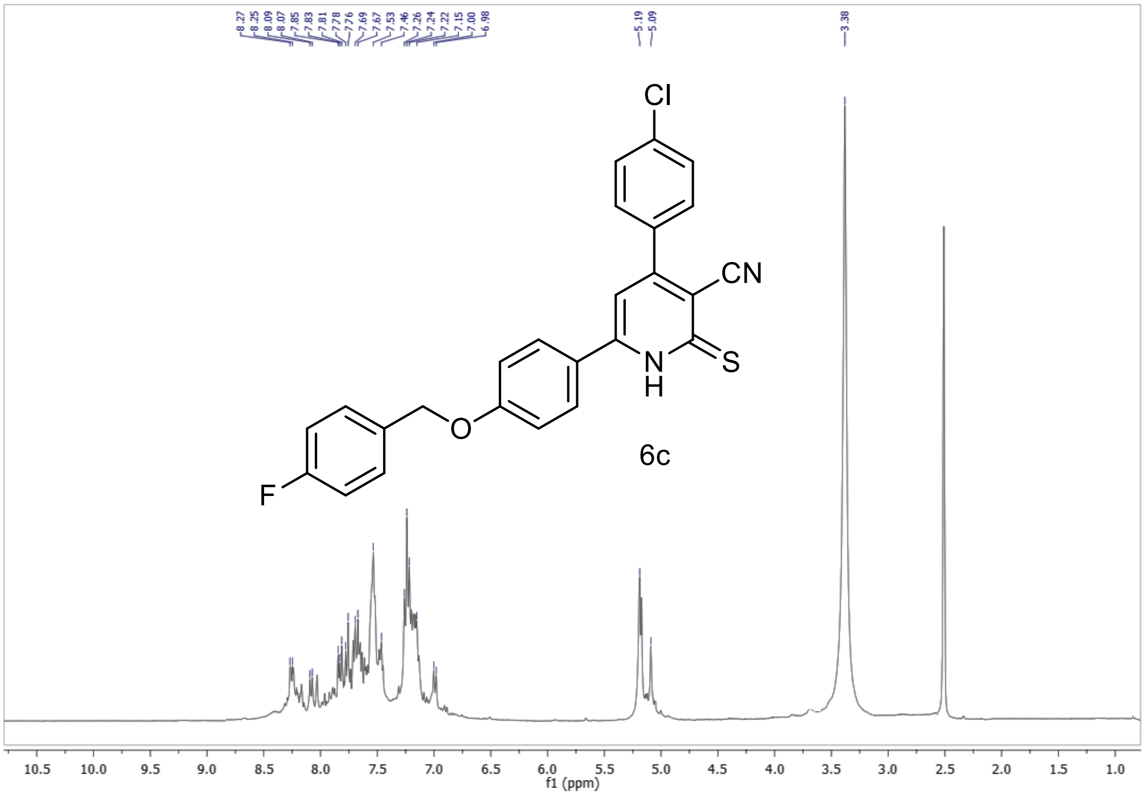

Figure S31. ^{1}HNMR spectrum of **6c**

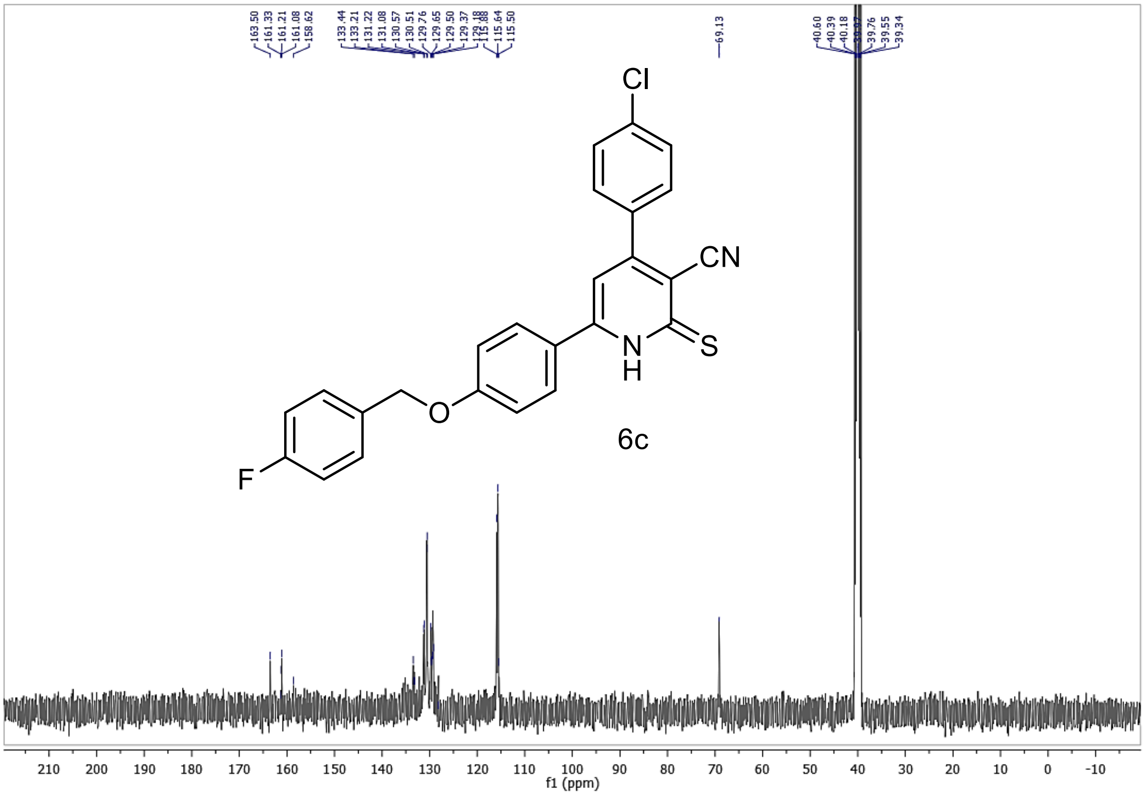

Figure S32. ^{13}CNMR spectrum of **6c**

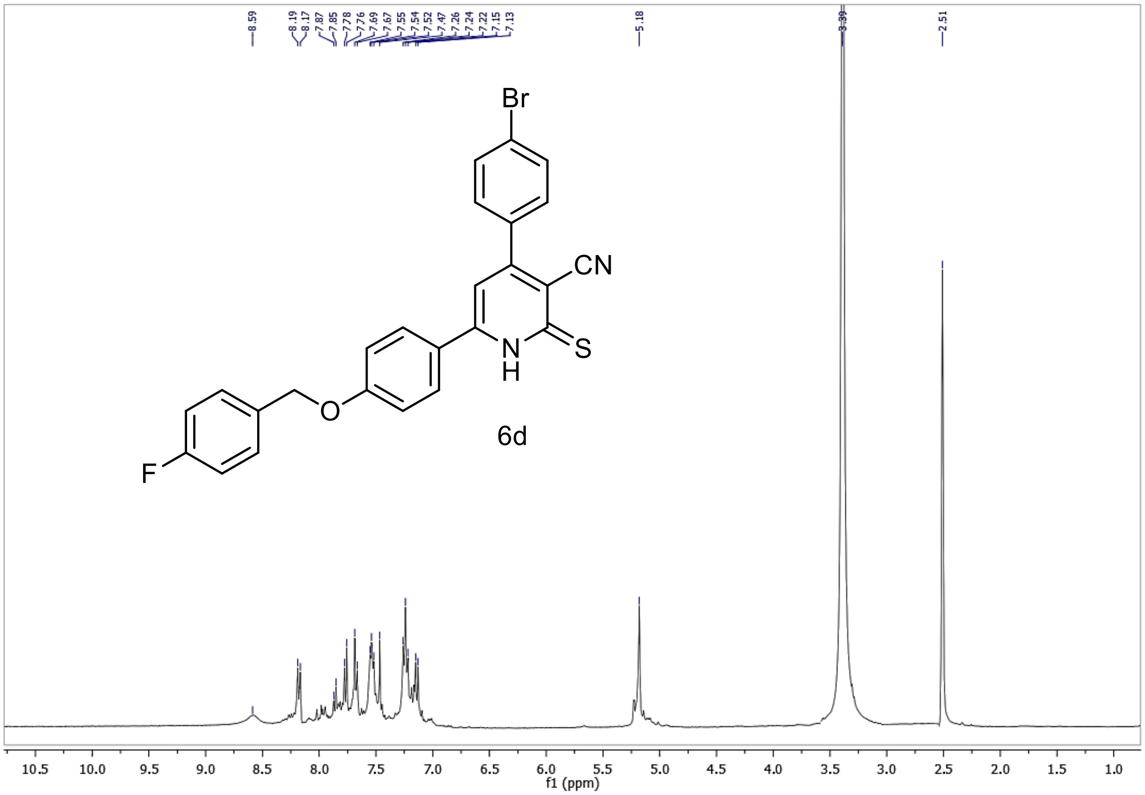

Figure S33. ^{1}HNMR spectrum of **6d**

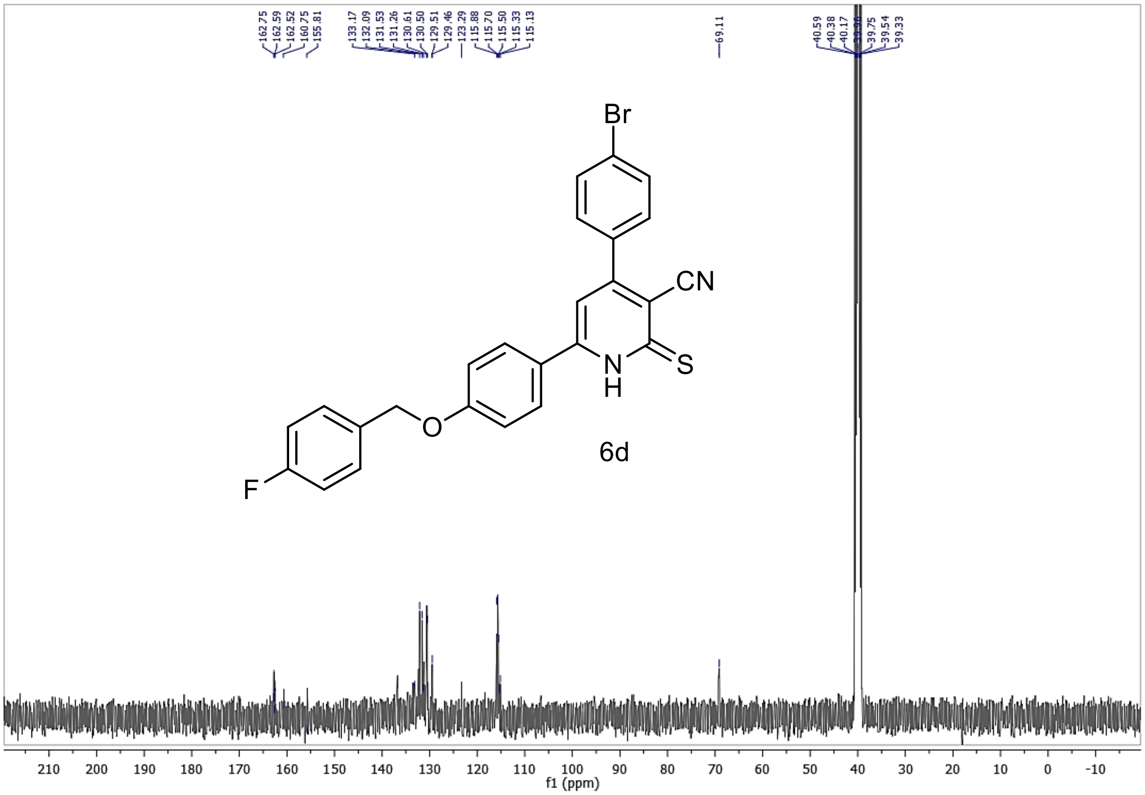

Figure S34. ^{13}CNMR spectrum of **6d**

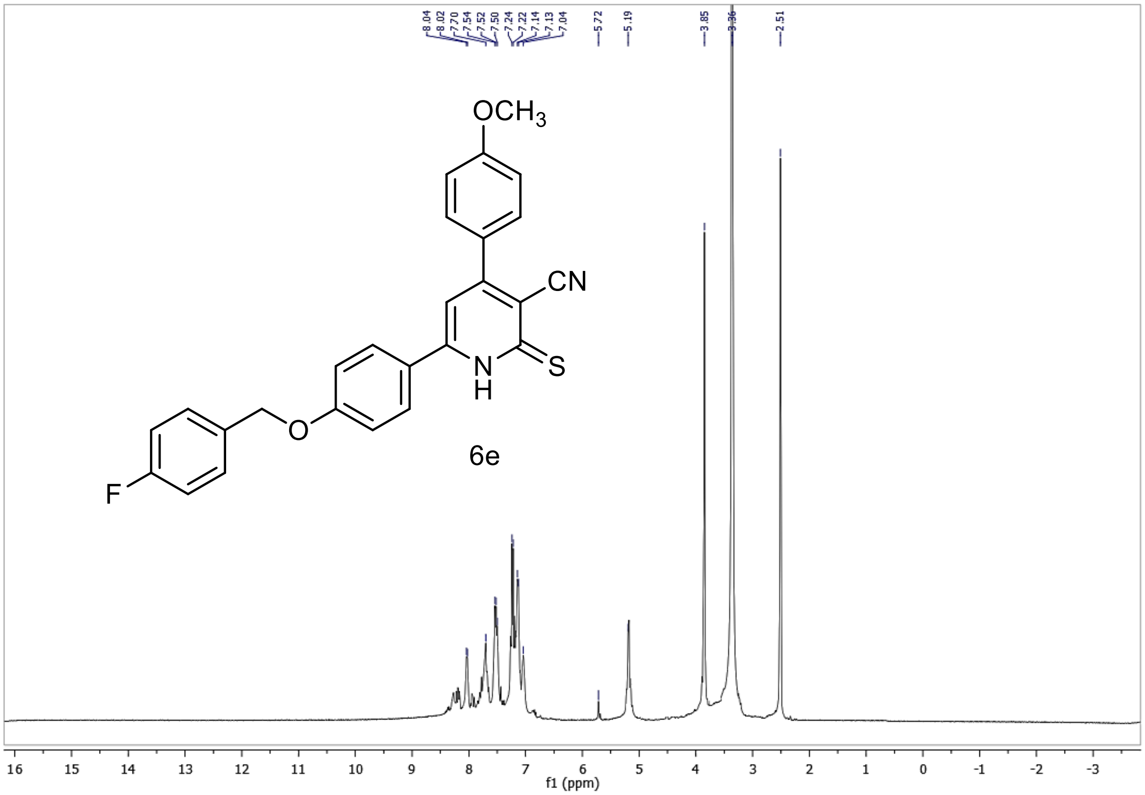

Figure S35. ^{1}HNMR spectrum of **6e**

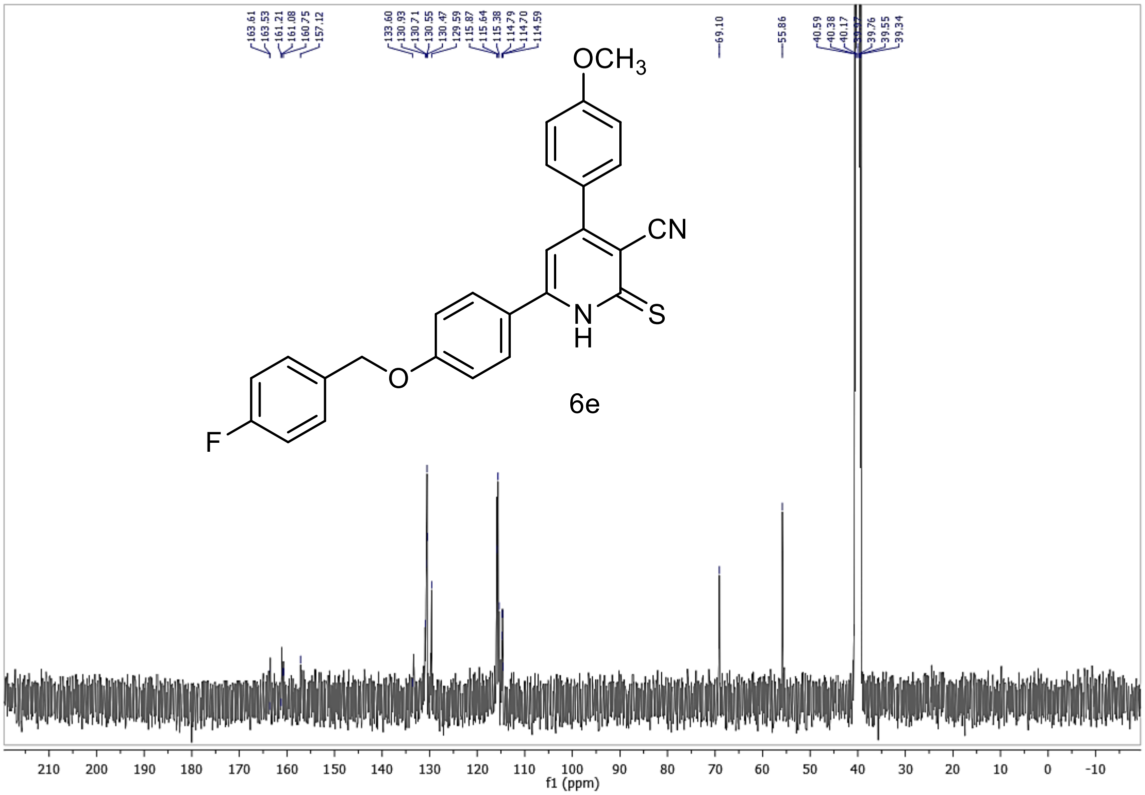

Figure S36. ^{13}CNMR spectrum of **6e**

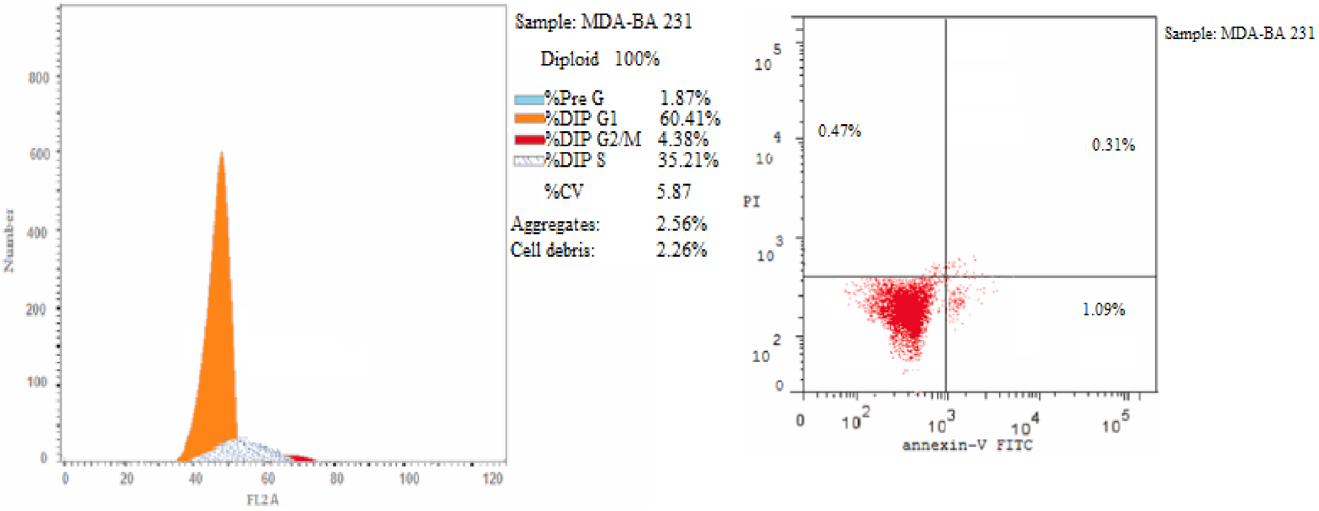

Figure S37. Cell cycle analysis and Annexin V-FITC apoptosis assessment for control MDA-BA 231.

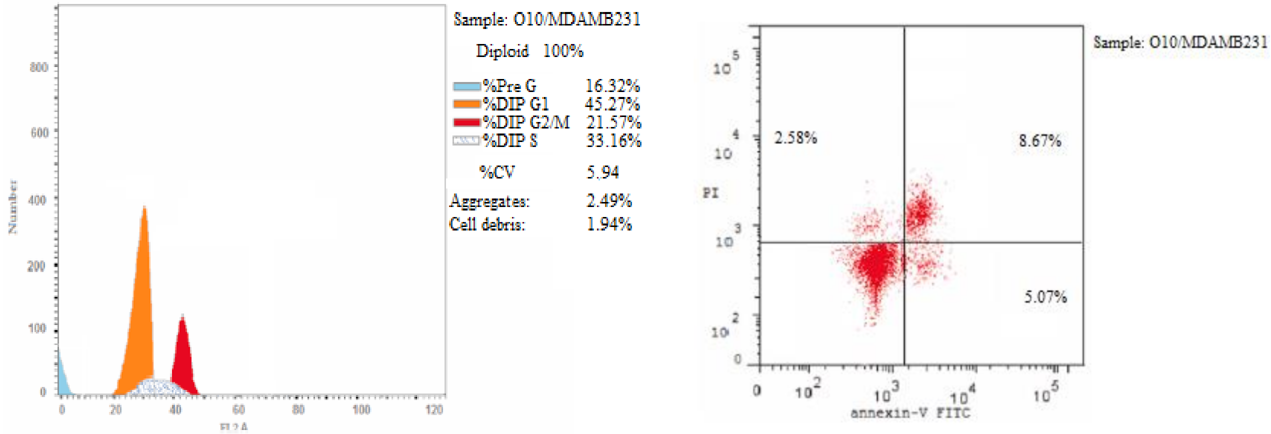

Figure S38. Cell cycle analysis and Annexin V-FITC apoptosis assessment for compound **5c**.

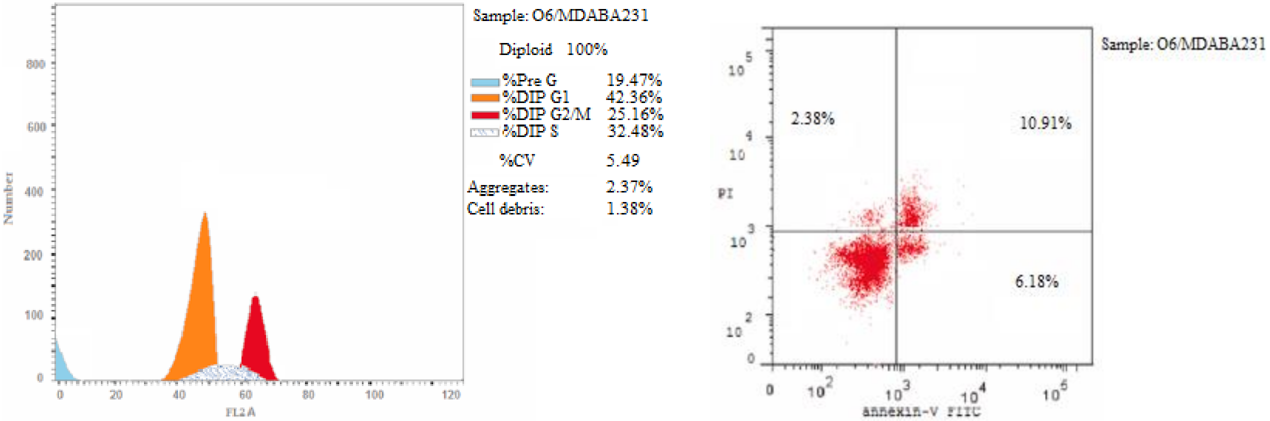

Figure S39. Cell cycle analysis and Annexin V-FITC apoptosis assessment for compound **5e**.

A

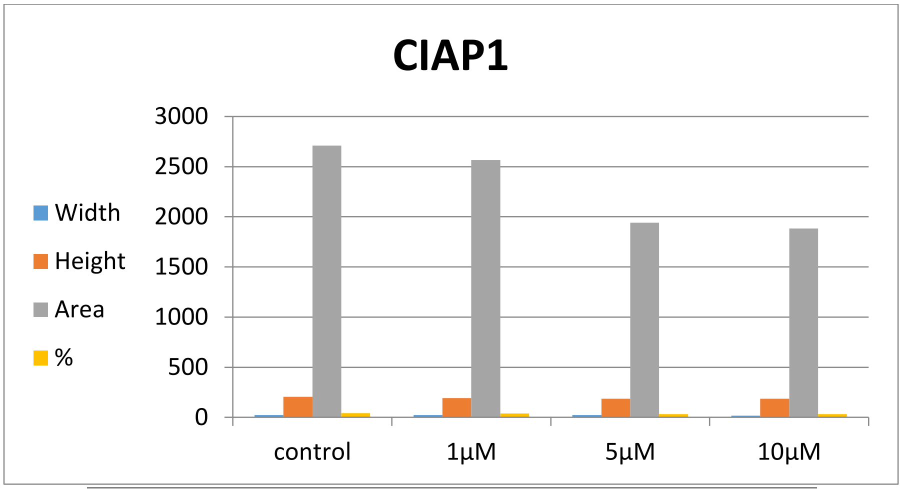

	Width	Height	Area	%
**control**	24	205	2709	41.2
**1µM**	24	192	2565	39.7
**5µM**	22	187	1941	34.1
**10µM**	18	186	1884	31.9

B

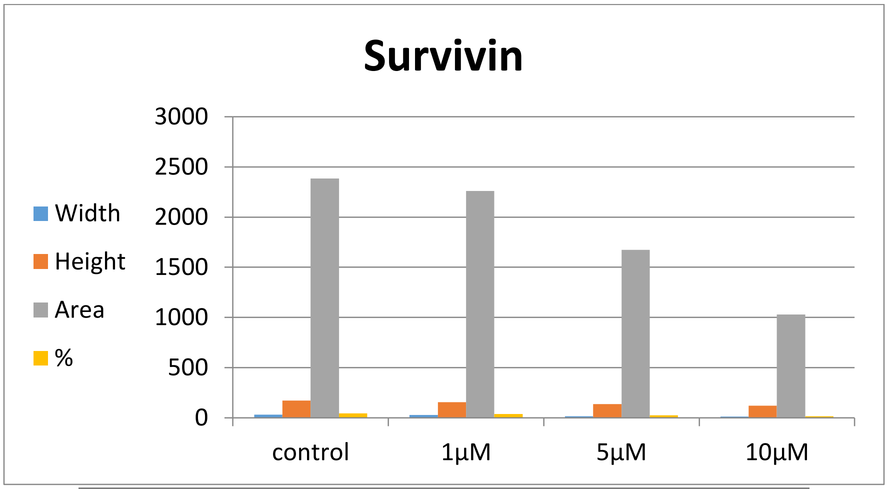

	Width	Height	Area	%
**control**	32	172	2384	41.9
**1µM**	26	156	2261	38.3
**5µM**	16	137	1674	25.9
**10µM**	13	121	1028	15.6

C

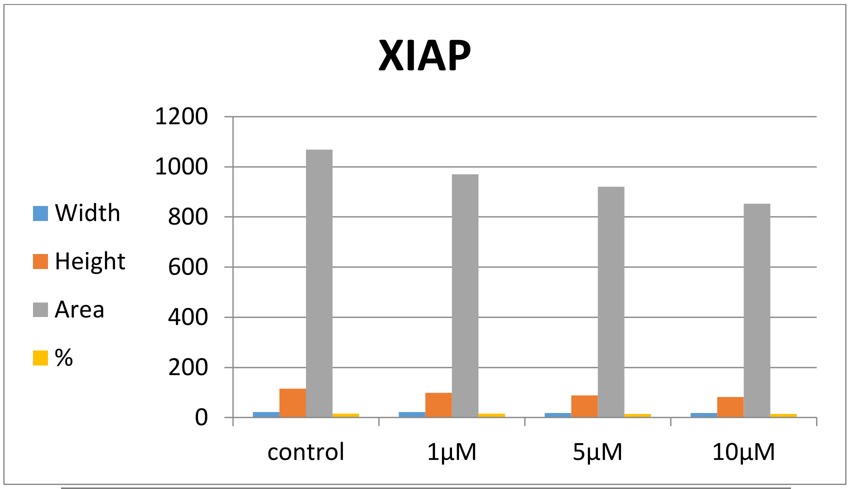

	Width	Height	Area	%
**control**	22	115	1068	16.4
**1µM**	22	99	970	16.3
**5µM**	19	88	921	15.1
**10µM**	19	82	853	14.2

D

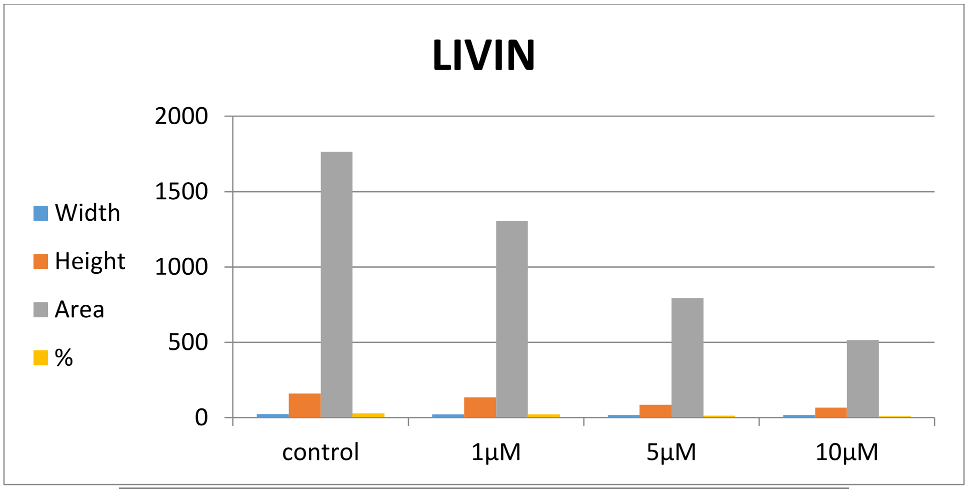

	Width	Height	Area	%
**control**	23	159	1765	26.9
**1µM**	22	134	1305	20.2
**5µM**	17	84	793	13.4
**10µM**	17	66	514	9.1

Figure S40. Western blotting assay analysis for **5c**; A: CIAP, B: Survivin, C: XIAP, D: Livin

A

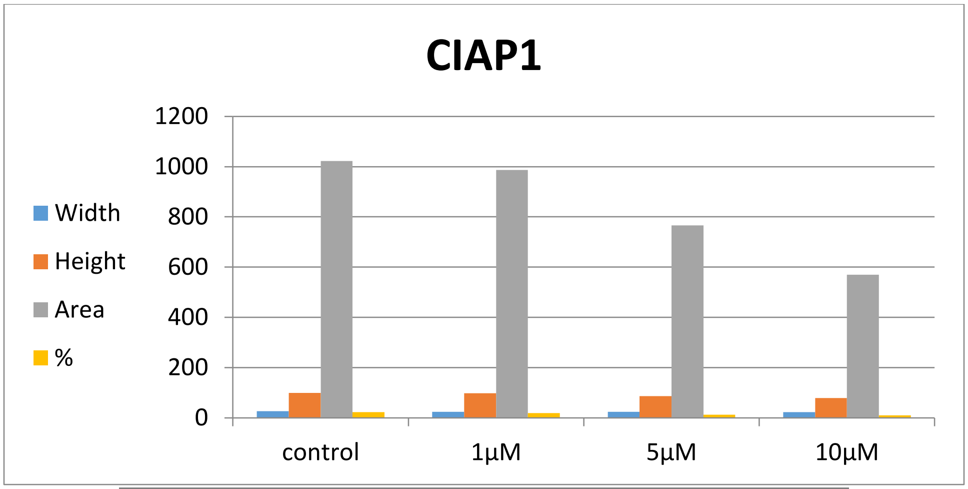

	Width	Height	Area	%
**control**	26	99	1022	22.8
**1µM**	24	98	987	18.2
**5µM**	23	86	766	12.6
**10µM**	22	79	570	10.2

B

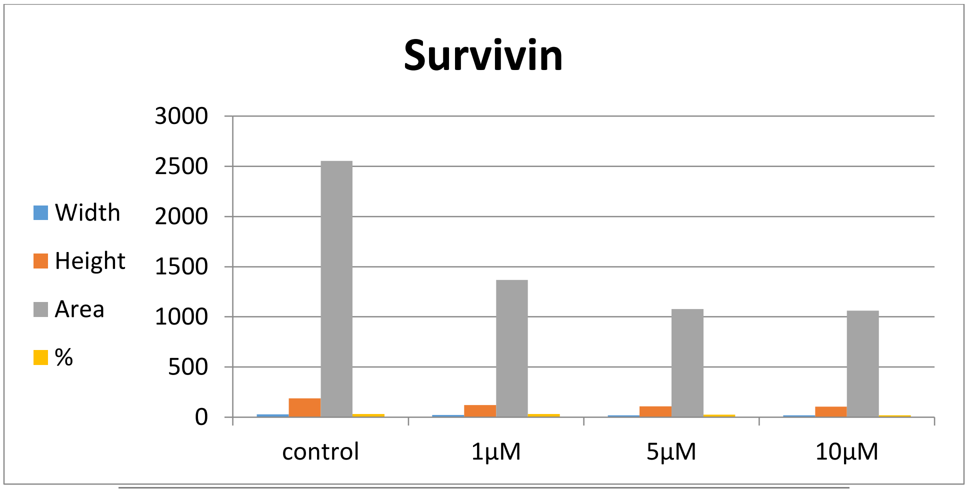

	Width	Height	Area	%
**control**	28	186	2554	32.2
**1µM**	22	120	1368	31.4
**5µM**	19	108	1079	25
**10µM**	18	105	1062	19.1

C

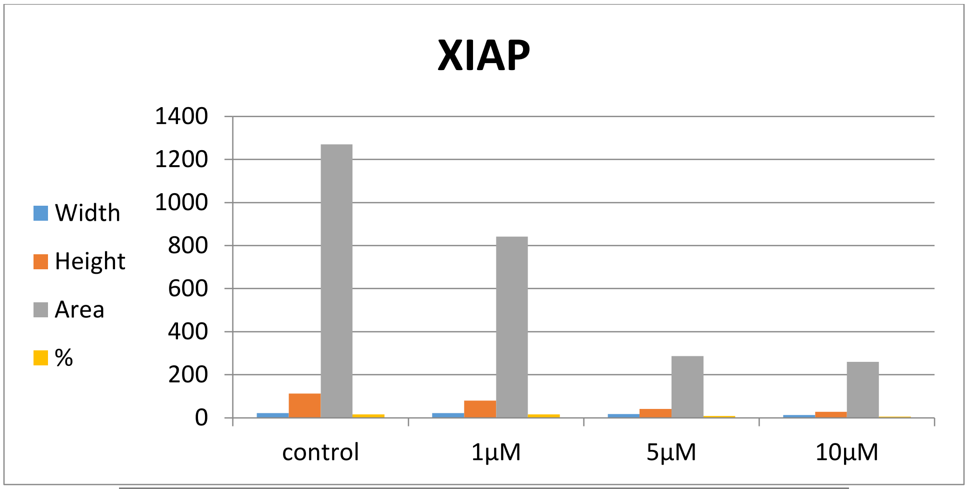

	Width	Height	Area	%
**control**	22	112	1270	15.6
**1μM**	22	80	841	15.4
**5μM**	17	41	287	8.6
**10μM**	13	28	260	4.7

D

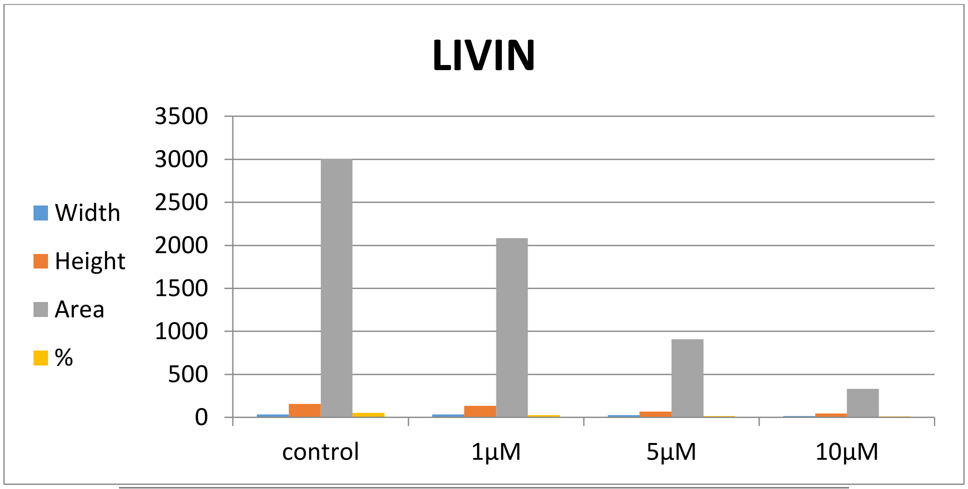

	Width	Height	Area	%
**control**	33	158	3002	53.8
**1μM**	33	136	2085	25.7
**5μM**	27	68	907	16.6
**10μM**	14	44	330	9.8

Figure S41. Western blotting assay analysis for **5e** A: CIAP, B: Survivin, C: XIAP, D: Livin
